# Synthetic cytokine circuits that drive T cell infiltration into immune-excluded tumors

**DOI:** 10.1126/science.aba1624

**Published:** 2022-12-16

**Authors:** Greg M. Allen, Nicholas W. Frankel, Nishith R. Reddy, Hersh K. Bhargava, Maia A. Yoshida, Sierra R. Stark, Megan Purl, Jungmin Lee, Jacqueline L. Yee, Wei Yu, Aileen W. Li, K. Christopher Garcia, Hana El-Samad, Kole T. Roybal, Matthew H. Spitzer, Wendell A. Lim

**Affiliations:** 1 Department of Medicine, UCSF; San Francisco, United States.; 2 Cell Design Institute; UCSF, San Francisco, United States.; 3 Department of Cellular and Molecular Pharmacology, UCSF; San Francisco, United States; 4 Biophysics Graduate Program, UCSF; San Francisco, United States; 5 Department of Microbiology and Immunology, UCSF; San Francisco, United States; 6 Department of Molecular and Cellular Physiology and Structural Biology, Howard Hughes Medical Institute, Stanford University; Stanford, United States; 7 Parker Institute for Cancer Immunotherapy, UCSF; San Francisco, United States; 8 Department of Otolaryngology-Head and Neck Surgery, UCSF; San Francisco, United States; 9 Helen Diller Family Comprehensive Cancer Center, UCSF; San Francisco, United States; 10 Department of Biochemistry and Biophysics, UCSF; San Francisco, United States

## Abstract

CAR T cells are ineffective against solid tumors with immunosuppressive microenvironments. To overcome suppression, we engineered circuits in which tumor-specific synNotch receptors locally induce production of the inflammatory cytokine, interleukin-2 (IL-2). These cytokine delivery circuits can potently enhance CAR T cell infiltration and clearance of immune-excluded tumors (immunocompetent models of pancreatic cancer and melanoma) without systemic toxicity. The most effective IL-2 induction circuit acts in an autocrine and TCR/CAR-independent manner, bypassing suppression by host cells that either consume IL-2 or inhibit TCR signaling. These engineered autocrine cells are able to establish an effective foothold in the tumors, likely because synNotch-induced IL-2 production can cooperatively enable initiation of CAR-mediated T cell expansion and killing. Thus, it is possible to reconstitute synthetic T cell circuits that activate the outputs ultimately required for a robust anti-tumor response, but in a manner that evades key points of tumor suppression.

## One-Sentence Summary

Synthetic circuits that deliver IL-2 locally to tumors allow CAR-T cells to overcome suppressive tumor microenvironments.

Chimeric antigen receptor (CAR) T cells have demonstrated remarkable success in the treatment of B cell malignancies (1, 2). Nonetheless, application of CAR or T cell receptor (TCR) engineered T cells to solid tumors has proven far more challenging (3). Many solid tumors create an immune-excluded local microenvironment that blocks the infiltration, activation, or expansion of cytotoxic T cells (4). Within this tumor microenvironment, activation of CAR/TCR pathways are inhibited by local immunosuppressive factors and cells (5–7). While evidence suggests that local administration of high-dose inflammatory cytokines could help reverse tumor suppression (8), combining adoptively transferred T cells with systemic cytokine administration or engineered cytokine production has shown either systemic toxicity or poor efficacy (9–11). There is a clear need to engineer next-generation therapeutic T cells with an enhanced ability to overcome tumor suppression, without exacerbating off-target or systematic toxicity.

**Fig. 1.**
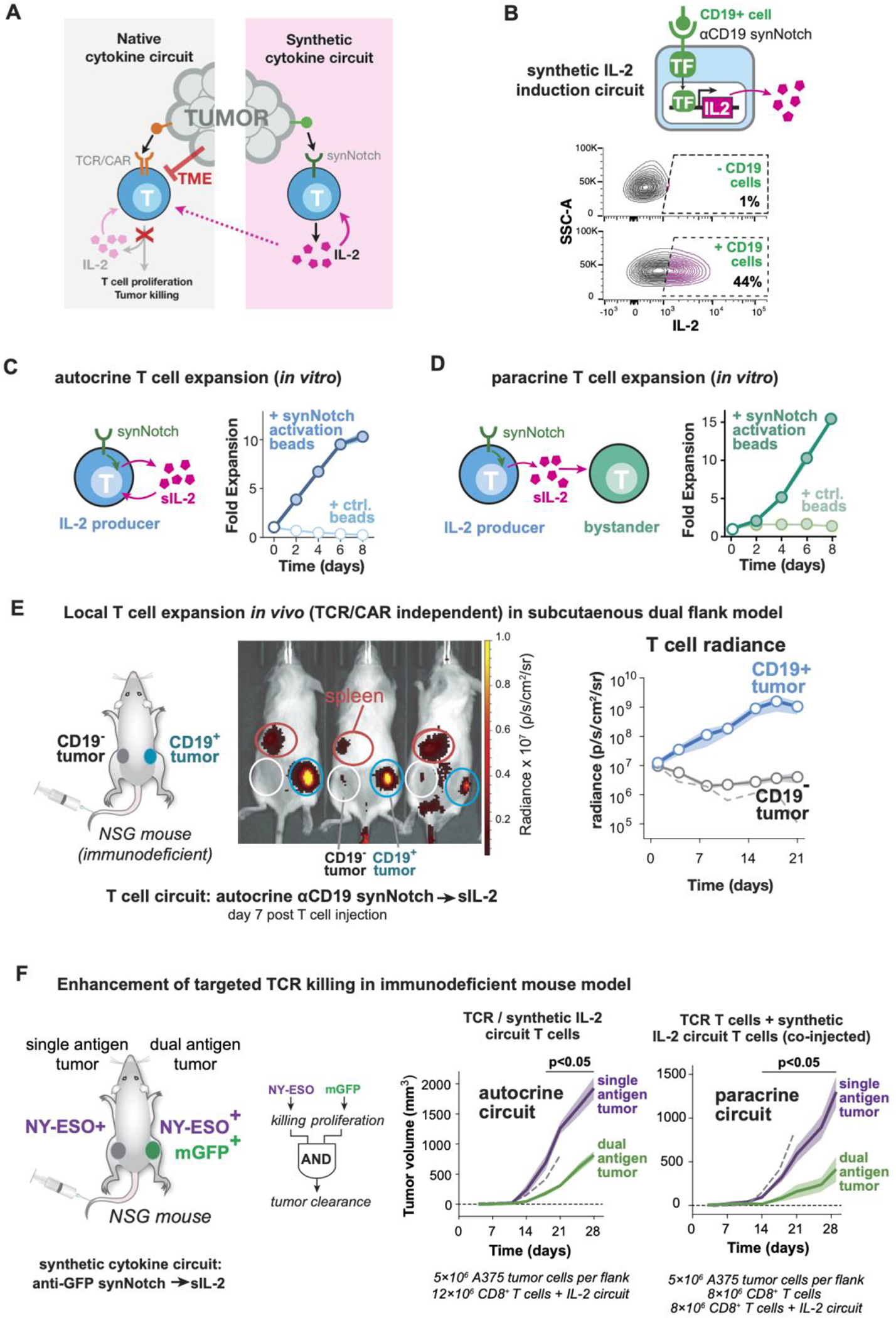
Synthetic synNotch→IL-2 circuits can drive local T cell proliferation independent of TCR activation or cooperatively with T cell killing. (**A**) The tumor microenvironment (TME) acts to suppress T cell activation, including inflammatory cytokine (e.g. IL-2) production. To bypass suppression, we propose to engineer synthetic IL-2 circuits trigged by tumor antigens in a manner independent from TCR/CAR activation. (**B**) Synthetic IL-2 circuits were created in human primary T cells using anti-CD19 synNotch receptors to drive production of an inflammatory cytokine (super IL-2/sIL-2). IL-2 is produced only when stimulated by A375 tumor cells bearing the cognate CD19 antigen. Compare to Figure S1A. (**C**) Synthetic IL-2 circuit drives autocrine proliferation of primary human T cells *in vitro*, only when the circuit is triggered (here myc-tagged synNotch is activated by anti-myc antibody coated beads). (**D**) Synthetic IL-2 circuit signals in a paracrine fashion to stimulate proliferation of a bystander population of human T cells that lack a synthetic circuit *in vitro*. For *C and D,* median is plotted; shading shows S.E.M., n=3 and filled markers indicate significant expansion > 1, right-tailed students t-test, p <0.05. Additional replicates of autocrine and paracrine proliferation are in Figure S1B. (**E**) Dual flank tumor model in NSG mice to monitor T cell trafficking *in vivo*. Primary human T cells were engineered with synthetic anti-CD19→sIL-2 circuit and eff-luc (to track cells) and administered to mice engrafted with CD19^+^ (right) and CD19^−^ (left) K562 tumors. Example bioluminescence imaging shown 7 days after T cell injection. Circles indicate tumors (blue, white) and spleen (red). Plot shows quantification of T cell luminescence over time for CD19^+^ and CD19^−^ tumors. Dashed line shows T cells in CD19^*+*^ tumor with no circuit added; shading shows S.E.M. (**F**) Tumor reactive T cells, such as ones bearing an anti-NY-ESO TCR, fail to produce effective cytokine and killing responses against antigen positive tumors. We hypothesize that simultaneously engaging the TCR and a synthetic IL-2 circuit could enhance a local T cell response. In this case T cells bearing an anti-NY-ESO TCR and an anti-membrane-bound GFP (mGFP) synNotch→sIL-2 circuit could function as an AND gate that requires two antigen inputs to stimulate tumor killing allowing more precise recognition strategies. Here a two-flank A375 tumor model in NSG mice, with NY-ESO only on left and NY-ESO/GFP on right was generated. Plots show tumor growth over time. Both autocrine and paracrine forms of the TCR + anti-GFP synNotch→sIL-2 cells show significantly enhanced control of only the dual antigen tumor. Error shading: S.E.M. Dashed line indicates dual antigen tumor growth curve with no T cell treatment. NY-ESO TCR only control and individual tumor growth curves available in Figure S4.

Here, we have created synthetic cytokine circuits as a strategy to improve therapeutic T cell activity against immune-excluded solid tumors. Using the recently developed synthetic Notch (synNotch) receptor (12, 13), we have created a bypass signaling pathway in which tumor recognition by synNotch induces local interleukin-2 (IL-2) production (Fig. 1A). The inflammatory cytokine IL-2 plays a critical role as both an output of T cell activation, and as a promoter of T cell activation and expansion (14–17). Suppressive tumor microenvironments can both reduce IL-2 production and/or competitively consume IL-2 (18–20). Thus, we hypothesized that providing IL-2 in a tumor-targeted, but TCR/CAR-independent manner, could help bypass tumor immune suppression. Indeed, we find that certain synthetic IL-2 circuits drive highly efficient CAR T cell infiltration and tumor control in immune-excluded solid tumor models, without concomitant systemic or off-target toxicity. Immune profiling shows expansion of CAR T cells only within the tumor, with increased markers of activation and decreased markers of exhaustion. Synthetic IL-2 production likely enables infiltrating T cells to survive and initiate sustained CAR-mediated activation, expansion and tumor killing. This type of synthetic cytokine delivery circuit could provide a powerful general approach for remodeling and overcoming immunosuppressive solid tumors.

## Results:

### Engineering synthetic IL-2 circuits that drive local T cell proliferation independent of T cell activation

To design a tumor-induced synthetic IL-2 circuit in T cells, we used a synNotch sensor to induce the transcription of an IL-2 transgene (Fig 1B). Briefly, synNotch receptors are chimeric receptors with a variable extracellular recognition domain, a Notch-based cleavable transmembrane domain, and an intracellular transcriptional domain (12, 13). Antigen binding induces intramembrane receptor cleavage, releasing the transcriptional domain to enter the nucleus and promote expression of a target transgene.

We built a prototype circuit in primary human T cells, using a synNotch receptor that recognizes the model antigen CD19, combined with a synNotch-responsive promoter driving expression of human IL-2 or an affinity-enhanced variant of IL-2 (known as super-2 or sIL-2) (21). As intended, stimulation of the synNotch receptor *in vitro* induced strong proliferation of the engineered cell population (Fig 1C). Cells with the anti-CD19 synNotch→sIL-2 circuit could function in a paracrine manner, driving the proliferation of co-cultured non-engineered T cells (Fig 1D) or NK cells (Fig S1C) *in vitro*. The degree of proliferation was dependent on the type of gamma-chain cytokine payload, with significant T cell proliferation seen with production of either IL-2 or sIL-2 (Fig S1D). Production of the homeostatic cytokine IL-7 (22) led to T cell survival with minimal expansion, while un-tethered IL-15 (23) had no effect. Thus, *in vitro*, a synNotch→sIL-2 circuit T cell can drive its own proliferation, as well as the proliferation of other co-cultured IL-2 responsive cells.

We then tested whether the synNotch→sIL-2 circuit could drive targeted expansion of human T cells *in vivo*, independent of CAR or TCR activation. We established a bilateral K562 tumor model in immunocompromised NOD *scid* gamma (NSG) mice, where only one flank tumor expressed the synNotch target antigen, CD19 (Fig 1E). Human primary CD8+ T cells engineered with the anti-CD19 synNotch→sIL-2 circuit were tagged with enhanced firefly luciferase (eff-luc) and injected intravenously. Cells with the synthetic IL-2 circuit autonomously identified the target tumor (CD19^+^/right) and locally expanded approximately 100-fold within this tumor (Fig 1E). In contrast, no off-target expansion was seen in the contralateral (CD19^−^) tumor. Flow cytometry analysis of tumor infiltrating lymphocytes (TILs) in the target and off-target tumor showed synNotch activation, T cell expansion, and proliferation only in the CD19+ tumor (Fig S2A–C). The administered T cells have no CAR or TCR reactivity against tumors, thus the synthetic production of IL-2 alone did not result in killing of the K562 tumors in this immunodeficient NSG mouse model (Fig S2D).

We also found that the anti-CD19 synNotch→sIL-2 circuit was also capable of driving T cell expansion in a paracrine (two-cell type) configuration, in this NSG mouse model. Here we co-injected a population of bystander T cells, which did not express the sIL-2 induction circuit but expressed luciferase to distinguish them from the synNotch→sIL-2 T cells. Co-injected into mice at a 1:1 ratio, the bystander cells also specifically expanded in the targeted (CD19^+^/right) tumor (Fig S3A–D) where the synNotch receptor was locally activated (Fig S3E). This paracrine T cell expansion was not observed in negative control experiments using synNotch T cells that either did not produce sIL-2 or did not recognize CD19 (Fig S3F).

In summary, this work represents one of the first examples in which locally targeted T cell expansion can be induced in a manner uncoupled from TCR or CAR activation.

### *Synthetic IL-2 circuits can enhance targeted T cell cytotoxicity* in vivo

Many engineered T cell therapies show effective cytotoxicity *in vitro* but fail to show sufficient proliferation or persistence to achieve effective tumor control *in vivo*. For example, cells bearing the affinity-enhanced anti-NY-ESO-1 TCR are able to lyse A375 melanoma tumors *in vitro* (24), but have shown limited clinical benefit in patients or preclinical models (25). We hypothesized that the addition of a synthetic cytokine circuit producing IL-2 might enhance tumor control by NY-ESO-1 T cells. Moreover, these T cells might function as a new type of AND gate (26, 27), where a therapeutic T cell exhibits enhanced specificity by requiring two antigens to be present before triggering its full cytotoxic response (the TCR antigen required for T cell activation, and the synNotch antigen required for inducing IL-2 production). In this case, we used an anti-GFP synNotch→sIL-2 synthetic cytokine circuit. By requiring the presence of both the TCR antigen (NY-ESO-1) and the synNotch antigen (in this case, membrane-tethered GFP) (Fig 1F), this cellular design strategy should further minimize off-target toxicity.

We examined the efficacy of anti-NY-ESO-1 TCR human T cells in NSG mice using a bilateral tumor model of a NY-ESO-1+ melanoma (A375). Only one flank tumor was co-labelled with the synNotch-targeted model antigen (membrane-tethered GFP). Anti-NY-ESO-1 TCR-expressing T cells lacking the synthetic IL-2 circuit were largely ineffective at controlling the growth of both the single (NY-ESO^+^) and dual (NY-ESO^+^/GFP^+^) antigen tumors (Fig S4A). However, when mice were treated with T cells simultaneously expressing both the anti-NY-ESO-1 TCR and the anti-GFP synNotch → sIL-2 circuit, the dual-targeted NY-ESO^+^/GFP^+^ tumor now showed a significant reduction in tumor size (Fig 1F). Similar tumor reduction was observed when IL-2 was provided in a paracrine configuration, by co-injection of one cell type only expressing the anti-NY-ESO-1 TCR and a second cell type only expressing the synthetic IL-2 circuit. Critically, in either the autocrine or paracrine configuration, the synthetic IL-2 circuit did not cause a reduction in the contralateral NY-ESO^+^/GFP^−^ tumor (lacking the synNotch ligand), highlighting the precisely targeted impact of the synthetic IL-2 circuit.

Using luciferase tracking of anti-NY-ESO-1 TCR T cells, we observed substantially increased intratumoral expansion of T cells only in tumors that were targeted by the synthetic IL-2 circuit (Fig S5A). The synthetic IL-2 circuit was only activated in the targeted double antigen positive tumor (Fig S5B), and we observed a significant increase in T cell activation markers in this targeted tumor (Fig S5C). A synthetic IL-2 circuit T cell without co-delivery of a tumor reactive cytotoxic T cell population did not produce tumor control in these NSG mouse models (Fig S5D).

### Autocrine configuration of synthetic IL-2 circuit is required in immunocompetent tumor models

**Fig. 2:**
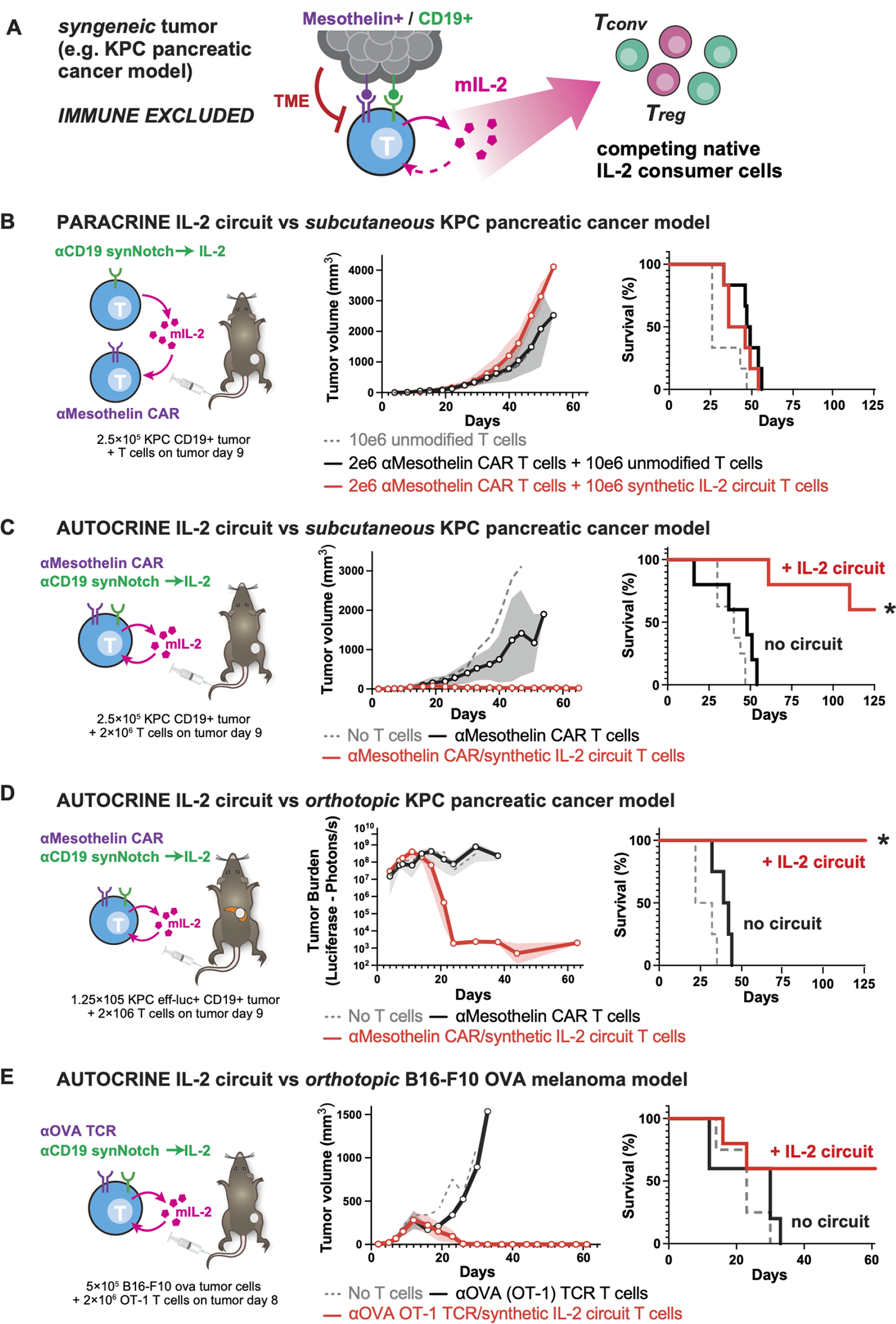
Autocrine synthetic IL-2 circuits strongly improve T cell cytotoxicity against multiple models of immune-excluded syngeneic tumors. (**A**) The synthetic IL-2 circuit was recapitulated in mouse T cells producing mouse IL-2 (mIL-2) to test circuits in presence of an intact immune system, suppressive TME and native IL-2 consumer cells. (**B**) KPC CD19^+^ pancreatic tumors were engrafted subcutaneously into immunocompetent C57/B16 mice and treated 9 days later with synthetic IL-2 circuit T cells and anti-Mesothelin CAR T cells as a two-cell paracrine system. No tumor control was observed in this paracrine configuration, even though KPC tumors express mesothelin. (**C**) KPC CD19^+^ pancreatic tumors were engrafted as in *B* and treated 9 days later with T cells engineered with both a synthetic IL-2 circuit and an anti-Mesothelin CAR (autocrine configuration). Significant improvement in tumor control was observed (*red lines*) compared to anti-Mesothelin CAR T cells combined with dummy synthetic cytokine circuit (synNotch only produces BFP, black lines). (**D**) KPC CD19^+^ pancreatic tumors were engrafted orthotopically in the pancreas tail and treated 9 days later with engineered T cells. 100% survival was observed only with the addition of the IL-2 circuit out to 120 days (duration of study). (**E**) B16F10 OVA CD19^+^ melanoma tumors were engrafted orthotopically into immunocompetent C57/B16 mice and treated 8 days later with 2e6 engineered mouse CD8^+^ OT-1 (anti-OVA) T cells. Tumor control was only observed in mice treated with T cells expressing the IL-2 circuit. For *B-E* All plots show tumor burden measured by average +/− S.E.M. of caliper or bioluminescence measurements and overall survival (n=4–5 per group, * = significant difference in survival with addition of IL-2 circuit using log-rank test, p < 0.05). See Fig. S7 and S8 for individual growth curves data.

Although the above results show that synthetic synNotch→ IL-2 circuits can significantly enhance T cell activity and expansion in immunodeficient mouse tumor models, we wanted to test whether they could also be effective in immunocompetent mouse models. Important factors influencing IL-2 production and consumption are likely missing in immunodeficient mouse models. Key missing factors include inhibitors of T cell activation (28) and the presence of competing IL-2 consumer cells (e.g. both native T cells, and T regulatory cells), which could significantly lower the effectiveness of synthetically produced IL-2 within tumors (29, 30). To study the effects of local IL-2 production within fully immunocompetent mouse tumor models, we rebuilt our synthetic IL-2 circuit in primary mouse T cells (Fig 2A). Primary CD3^+^ mouse T cells were engineered to express an anti-human-CD19 synNotch → mouse IL-2 (mIL-2) circuit. This circuit resulted in synNotch-induced proliferation of mouse T cells *in vitro*, just as was observed previously with human T cells (Fig S6A).

We then chose to deploy this IL-2 circuit in targeting the mouse pancreatic tumor model KPC (KrasLSL.G12D/+; p53R172H/+; PdxCretg/+) (31, 32), as this immune-excluded tumor exhibits the challenging immunotherapy refractory features of pancreatic ductal adenocarcinoma (PDAC) (33). Like most pancreatic ductal adenocarcinomas, these cells express the tumor target antigen mesothelin (34). Although anti-mesothelin mouse CAR T cells show robust cytotoxicity against KPC cells *in vitro* (Fig S6B), they show limited to no tumor control of KPC tumors *in vivo* (Fig S6C). Thus, this immune competent mouse model replicates the poor *in vivo* therapeutic efficacy reported in early phase clinical trials of standard anti-mesothelin CAR T cells in pancreatic cancer (3), making it an ideal model in which to test enhancement of the CAR T cells with synthetic IL-2 circuits. We engineered KPC tumor cells that, in addition to endogenously expressing the CAR antigen (mesothelin), also expressed a model synNotch antigen (human CD19).

We first tested CAR T cell enhancement by a paracrine synNotch→mIL-2 circuit. Anti-mesothelin CAR T cells were co-injected with a second T cell population expressing the anti-CD19 synNotch → mIL-2 circuit. Distinct from our studies in immunodeficient mice, these paracrine IL-2 circuit cells failed to improve tumor control in an immune competent context (Fig 2B, S7A). Instead, we found that in this fully immunocompetent tumor model, improved CAR T cell-mediated tumor control was only observed with the autocrine configuration of the synthetic IL-2 circuit – i.e. the cytotoxic receptor (CAR) and the synNotch→IL-2 circuit must be encapsulated within the same cell (Fig 2C, S7B). We hypothesize that the presence of competing host IL-2 consumer cells (e.g. bystander T cells and T_regs)_ in immune-competent models contributes to this major difference between the autocrine and paracrine circuits (i.e. paracrine circuits might be more sensitive to competing IL-2 sink cells), a model consistent with more in depth tumor profiling data in later sections of this paper.

The autocrine synthetic IL-2 circuit anti-Mesothelin CAR-T cells were extremely potent. In an even more challenging immune-competent mouse model, in which KPC tumors were engrafted orthotopically in the pancreas, complete tumor clearance was observed upon treatment (Fig 2D) —100% of mice survived, compared with 0% with CAR only T cells. Simply increasing the dose of anti-Mesothelin CAR-T cells had a negligible effect compared to addition of the synthetic IL-2 circuit (Fig S8A,B).

This type of autocrine IL-2 circuit also shows similar dramatic therapeutic improvement in treating a different type of immune-excluded solid tumor – B16-F10 OVA intradermal melanoma tumors, treated with OT-1 TCR expressing T cells (Fig 2E, S7C). Here again, OT-1 T cells without the cytokine circuit are ineffective *in vivo* in immune competent models (despite *in vitro* cytotoxic activity -- Fig S6D). Only when the OT-1 TCR is co-expressed with the autocrine synNotch→IL-2 circuit, do we observe effective infiltration and tumor clearance in the immune competent model.

### Comparison to other strategies of IL-2 co-delivery.

**Fig. 3.**
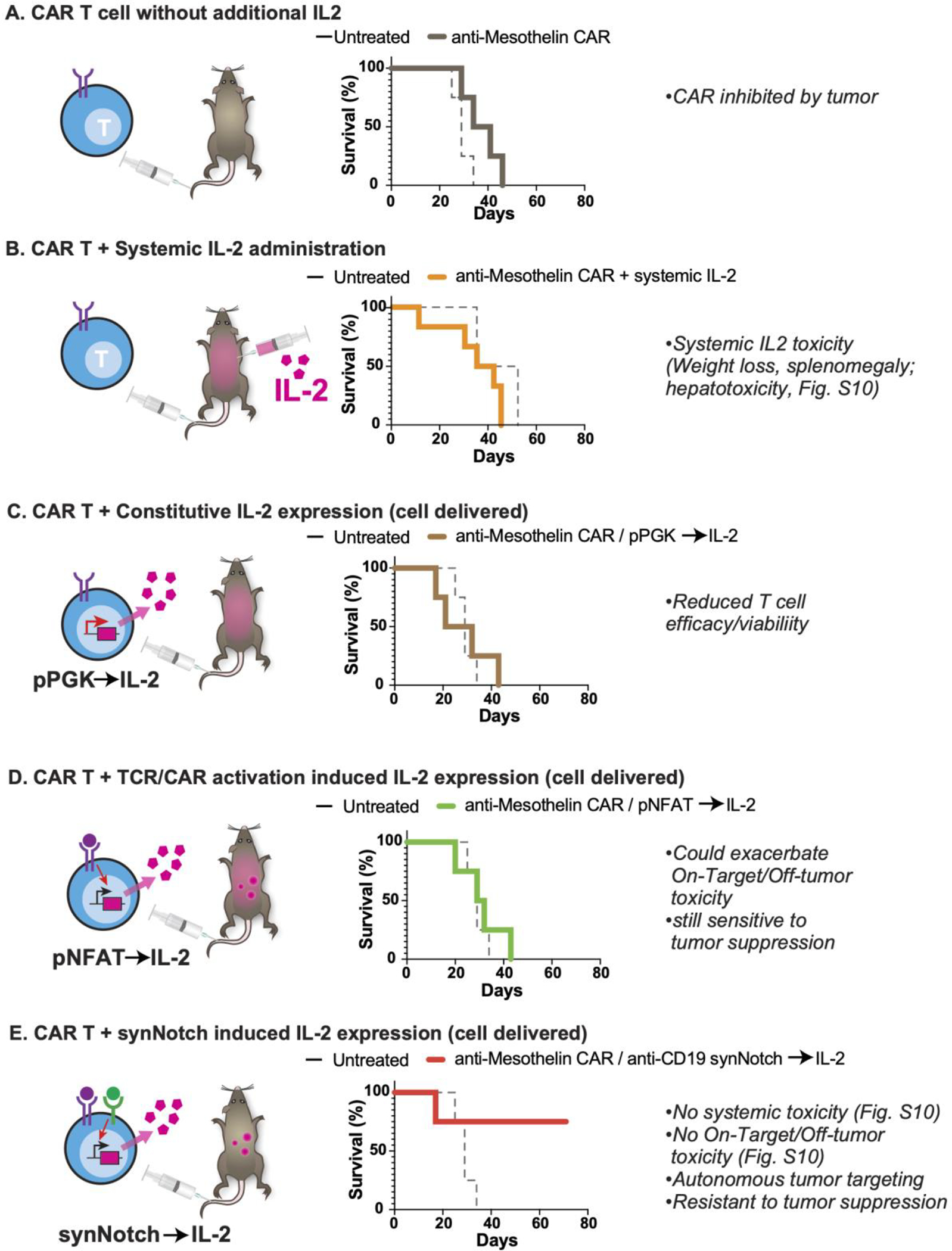
Synthetic Notch based cytokine delivery is required for effective control of KPC tumors. KPC CD19+ pancreatic tumors were engrafted subcutaneously into immunocompetent C57/Bl6 mice and treated 9 days later with T cells as labeled. Plotted is schematic for IL-2 production as well as overall survival for each cell design compared to matched untreated mice. n=4,5 per group. Tumor measurements for each condition are plotted in Figure S9. (**A**) 1e6 anti-Mesothelin CAR T cells with no additional IL-2. (**B**) 2e6 anti-Mesothelin CAR T cells with systemic IL-2 administered at high dose (250,000 to 750,000 IU/mL) twice daily intraperitoneally for 7 days. (**C**) 1e6 anti-Mesothelin CAR T cells engineered to constitutively express mIL-2 using a PGK promoter. (**D**) 1e6 anti-Mesothelin CAR T cells engineered to inducibly express mIL-2 under the control of a NFAT promoter. (**E**) 1e6 anti-Mesothelin CAR T cells engineered to inducibly express mIL-2 under the control of an anti-CD19 synNotch.

Importantly, this strong therapeutic improvement was not observed with other methods of co-delivering IL-2 with a CAR T cell. We tested systemic co-administration of IL-2 at maximum-tolerated doses (35) (Fig 3B, S9B), expression of IL-2 in the CAR T cell from a constitutive promoter (“armored CAR”) (Fig 3C, S9C), or expression of IL-2 from a T cell activated promoter such as pNFAT (36) (Fig 3D, S9D).

Systemically injected IL-2 led to systemic toxicity without improving CAR T cell activity (Fig S10B). Constitutive production of IL-2 was unable to support T cell proliferation *in vivo* (Fig S11A) likely in part due to significant silencing of the constitutive IL-2 transgene (Fig S11B) (37). IL-2 can have a biphasic effect on T cell survival (38) in part due to promotion of activation induced cell death (39) and T cell differentiation (40). We find that such negative effects are exacerbated by constitutive IL-2 production (Fig S11C). This suggests that when and how the IL-2 cytokine is produced is critical in determining the outcome.

Importantly, despite its potent anti-tumor efficacy, the synNotch→ IL-2 circuit showed no evidence of systemic cytokine toxicity or exacerbation of CAR T cell toxicity, as assessed by mouse survival, body weight, spleen weight, and measurements of hepatotoxicity (Fig S10). Moreover, the required recognition of two antigen inputs (CAR and synNotch antigens) should further enhance the specificity of tumor targeting (as seen by specific targeting to dual antigen tumor Fig S7C, and reduced hepatotoxicty S10C). In summary, combining a tumor-reactive TCR/CAR with an autocrine synNotch→IL-2 circuit, results in uniquely potent and localized anti-tumor enhancement.

### Synthetic IL-2 circuit drives T cell infiltration into immune excluded tumors

**Fig. 4.**
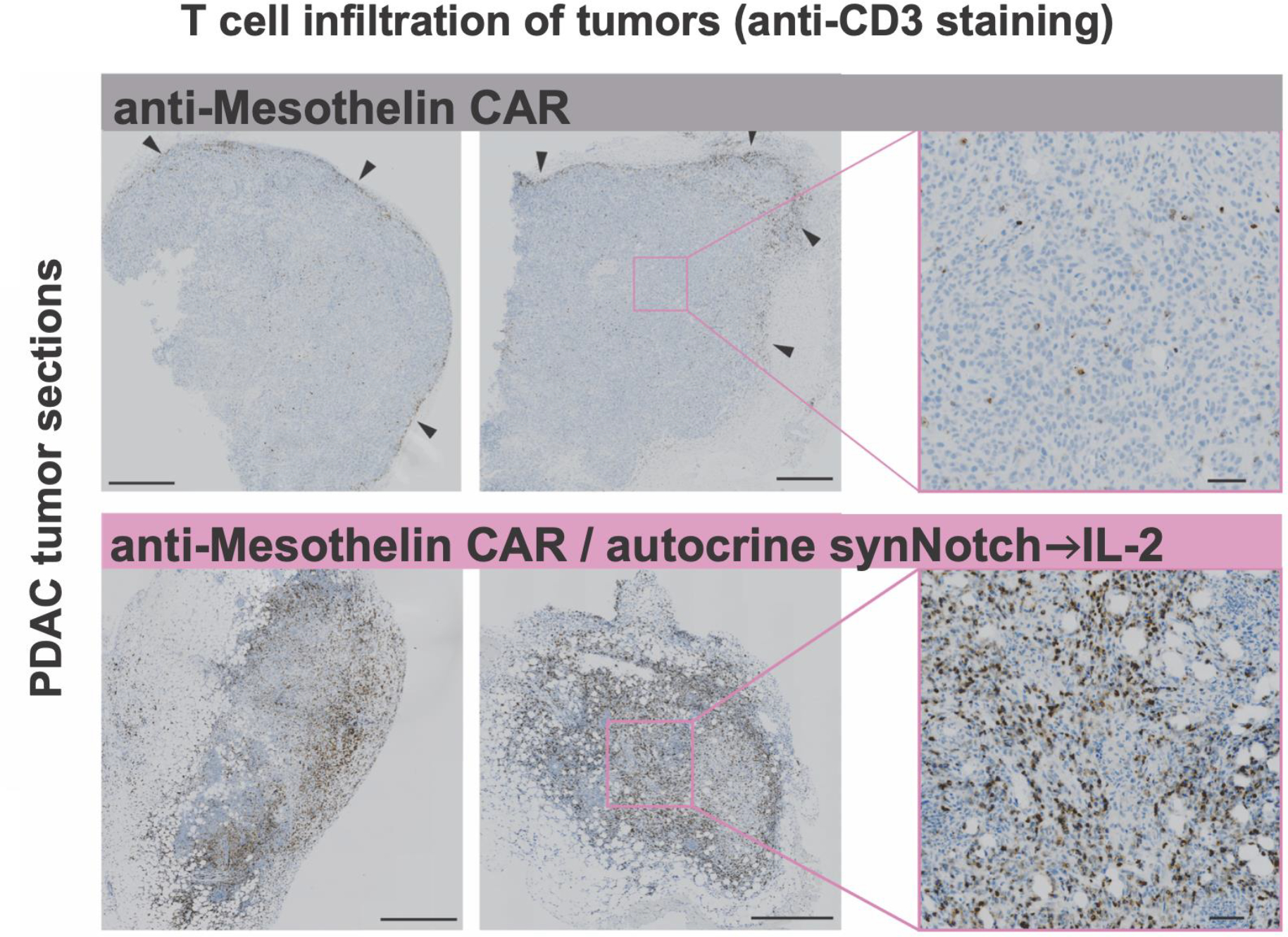
Synthetic IL-2 circuit enables T cell infiltration into immune excluded tumors. KPC CD19^+^ tumors were engrafted subcutaneously, treated with engineered T cells, and analyzed by IHC for T cell infiltration (anti-CD3 stain). Anti-mesothelin CAR T cells (*top*) failed to penetrate into the tumor, infiltrating the tumor edges (*black arrows*). Addition of synthetic autocrine IL-2 circuit (*bottom*) resulted in dramatically increased T cell infiltration into tumor core. Tumors were collected 23 days (*left*) and 8 days (*center*) after T cell injection. Zoomed out scale bars are 500 microns, zoomed in are 50 microns.

To better characterize how this autocrine synthetic IL-2 circuit improves CAR T cell control of syngeneic pancreatic tumor models, we profiled the tumors in more depth during treatment. We collected KPC pancreatic tumor specimens at the beginning and well into tumor regression (8 days and 23 days after T cell treatment) and measured CD3^+^ T cell infiltration using immunohistochemistry. Tumors treated with standard anti-mesothelin CAR T cells displayed a classic immune-excluded phenotype, with very limited T cell infiltrate inside the tumor core and most T cells gathered at the tumor periphery (Fig 4, top). In contrast, tumors treated with CAR T cells containing the synthetic autocrine IL-2 circuit showed substantially increased infiltration of T cells throughout the tumor core (Fig 4, bottom). A similar infiltration and expansion of the CD8+ lymphocytes also seen in B16-F10 OVA melanoma tumors sampled 10 days after treatment with OT-1 T cells bearing the synthetic IL-2 circuit (Fig S12A).

To profile the tumors in more detail, we performed flow cytometry and CyTOF analyses on excised and dissociated tumors. To track the endogenous (host) T cells independently from the adoptively transferred CAR T cells, we adoptively transferred congenic Thy1.1 or CD45.1 CAR T cells into Thy1.2 or CD45.2 mice, respectively, allowing us to clearly distinguish endogenous from transplanted T cells by FACS.

**Fig. 5.**
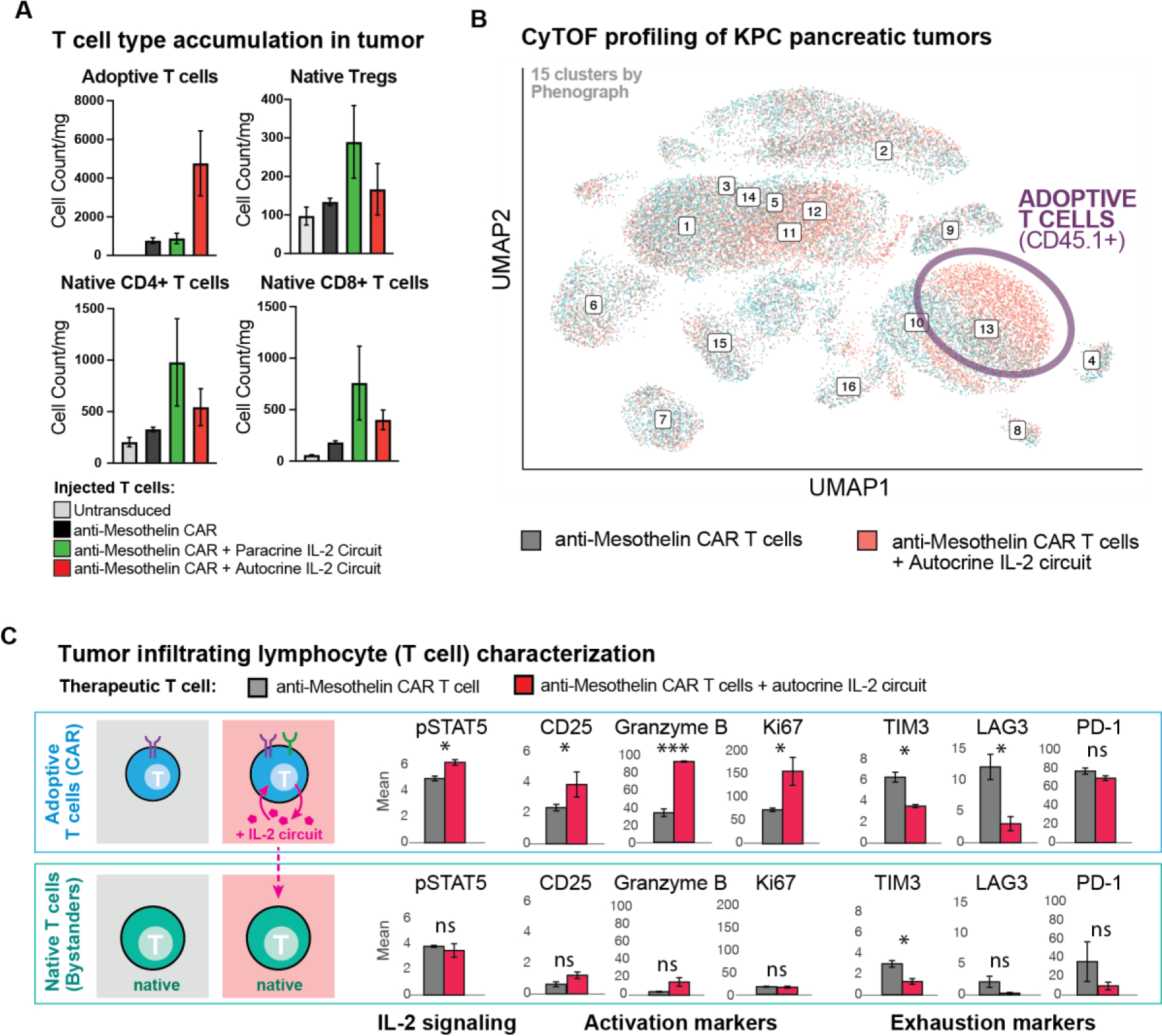
Profiling of tumor micro-environment shows expansion and activation of CAR T cells with autocrine IL-2 circuit. (**A**) Treated KPC CD19^+^ tumors were collected as in (*A*) after 9 days for analysis by CyTOF using CD45.1 as a marker of adoptively transferred T cells and CD45.2 as marker of native T cells. Native T cells and Regulatory T cells (Tregs) showed expansion in tumors treated with anti-mesothelin CAR + synthetic IL-2 circuit in autocrine or paracrine configuration, while adoptive (CAR) T cells showed far more dramatic expansion only with anti-mesothelin CAR + synthetic IL-2 circuit in autocrine configuration. n=3 samples per treatment, no p value calculated. Counts are normalized to tumor weight. (**B)** Unsupervised analysis of CyTOF data. UMAP shown for KPC tumors treated by anti-mesothelin CAR +/− IL-2 circuit (autocrine). Labelled numbers indicate clusters by Phenograph. Enrichment was only seen in adoptively transferred CAR T cells when the synthetic IL-2 circuit was engaged, see Fig S13 for mean marker expression for each Phenograph cluster, and measure of cluster enrichment. (**C**) Analysis of tumor infiltrating lymphocytes in markers in CAR T cells (CD45.1) from CyTOF data shows that CAR T cells with the synthetic IL-2 circuits in autocrine show higher expression of markers of IL-2 signaling (pSTAT5), activation (CD25), effector function (Granzyme B) and proliferation (Ki67), while showing decreased expression of markers of exhaustion (Tim3, Lag3, PD1). Matched analysis of native T cells (CD45.2) shows limited IL-2 signaling, activation, effector responses, proliferation, or exhaustion markers, with or without addition of synthetic IL-2 circuit. Mean +/− S.D. is plotted. See Fig S13–S15, for additional data including repeat CyTOF run. Statistical significance was tested using a two-tailed Student’s t test [not significant (ns) > 0.05, *P < 0.05, *** P < 0.001].

These studies showed that the engineered autocrine T cells (expressing both CAR and the synNotch→IL2 circuit) drove substantial intra-tumoral infiltration of both adoptively transferred (engineered) T cells and native host T cells (Fig 5A, S12B). In contrast parallel analysis of tumors treated with the paracrine synNotch→IL-2 circuit (CAR and synthetic cytokine circuit are expressed by two separate, co-injected cell types) showed expansion of native T cells only and no expansion of the adoptively transferred CAR T cells (Fig 5A, S13B), suggesting that in the paracrine configuration, induced IL-2 was primarily consumed by competing native T cells, leaving little available to drive expansion of the rarer CAR T cells.

Unsupervised clustering (41) of the CyTOF measurements (from the CD45^+^ immune cell infiltrate in KPC tumors) identified that the primary therapeutic effect of the autocrine IL-2 circuit was to enrich the population of activated adoptively transferred CAR T cells (Fig 5B). Little change was seen in the myeloid compartments (42), suggesting that synthetic IL-2 production acts primarily to drive T cell infiltration (both native and adoptive) and not by altering myeloid cell associated immune suppression. Furthermore, the expansion of T cells was completely constrained to the tumor - no changes were seen in immune cells from isolated spleens by flow cytometry or CyTOF analysis (Fig S12A, Fig S16), highlighting the focused local activity of the engineered cytokine circuit.

In addition to driving expansion of cytotoxic T cells in these immunologically cold tumors, the synthetic autocrine IL-2 circuit improved the phenotypes of the CAR T cells that infiltrate the tumor. CyTOF analysis showed that the synthetic autocrine IL-2 circuit upregulated markers of T cell activation (CD25), effector activity (Granzyme B) and proliferation (Ki67). Conversely, these IL-2 enhanced T cells also showed reduced expression of markers of exhaustion (Tim3, Lag3, PD-1) (Fig 5C, S14B) (43). Most native T cells (non-CAR) found in the tumors, however, appear to act simply as IL-2 sinks – they did not show markers of activation, effector function, proliferation, or exhaustion (Fig 5C, S14B), but instead largely exhibited a naïve phenotype (Fig S14C,D, S15C). The phenotype of the regulatory T cell population was mostly unchanged (Fig S14D, S15D). These findings suggest that the tumor has a significant population of native host T cells that, in bulk, compete to consume IL-2 without contributing to the anti-tumor response, (akin to T_reg_ suppression via IL-2 consumption).

## Discussion

### Cell delivered IL-2 is a powerful tool to synergize with therapeutic T cells

Cytokines such as IL-2 have long been known as powerful stimulators of anti-tumor immunity (44). However, systemic IL-2 delivery is also well known to be highly toxic, leading to a broad set of adverse effects including capillary leak syndrome, thereby greatly limiting its therapeutic use (45). Most current efforts in IL-2 engineering have focused on engineering the cytokine to be more selective for a tumor. Here instead we use a different strategy: harnessing the power of an engineered cell to identify a tumor and locally deliver IL-2 exactly where it is needed. We show that cell-mediated local cytokine (IL-2) delivery can effectively overcome immune suppression, augmenting CAR T cells to efficiently clear multiple immune-excluded tumor models (pancreatic cancer and melanoma) that are otherwise nearly completely resistant to standard CAR T cell treatment.

**Fig. 6.**
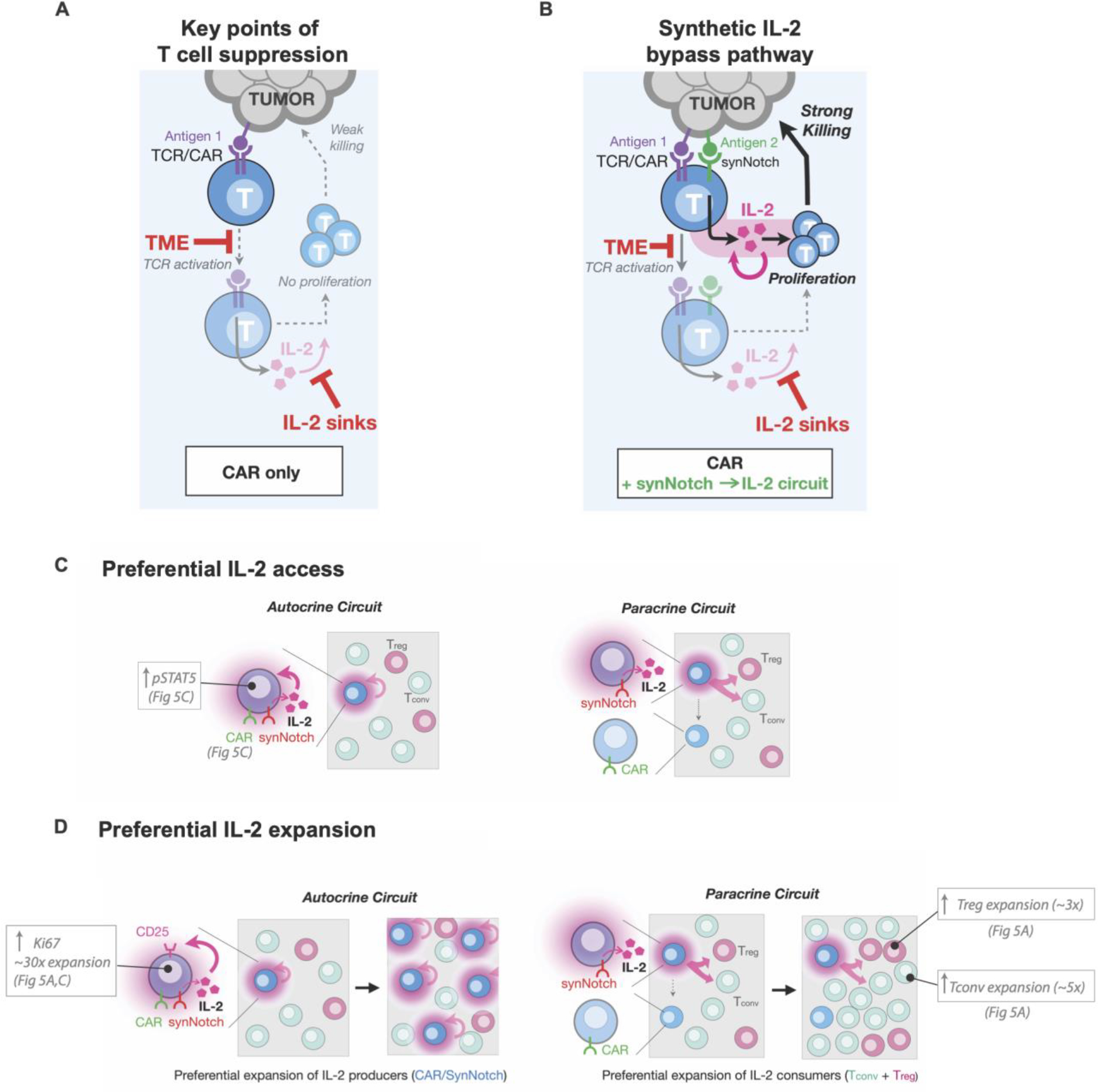
Bypassing tumor immune suppression mechanisms with a synthetic IL-2 delivery circuit (**A**) Standard CAR/TCR T cell activity in suppressive microenvironments is limited by inhibition of T cell activation, minimal production of IL-2, and consumption of IL-2 by competing native cells (sinks). Activation of both TCR and cytokine signaling, required for the full T cell response (AND gate), is blocked at these steps. (**B**) Creating a bypass channel for IL-2 production that is independent of CAR/TCR activation can overcome key suppressive steps. New circuits allow initiation of T cell activation via synergistic TCR/cytokine stimulation, leading to positive feedback, T cell activation, proliferation, and efficient killing of tumor cells. The synthetic circuit reconstitutes the key requirements for a strong T cell response in a manner that bypasses key suppressive bottlenecks. (**C**) Schematic differences between autocrine and paracrine IL-2 signaling in the presence of IL-2 consumers. An autocrine IL-2 circuit provides preferential spatial access to self-made IL-2 in comparison to a paracrine IL-2 circuit, where CAR T cells must compete with other IL-2 consumers (Tregs or T-naive cells). (**D**) An autocrine IL-2 leads to preferential expansion of IL-2 producers (through T cell activation and upregulation of CD25) in contrast in a paracrine circuit IL-2 producers compete on equal or lesser footing with IL-2 consumers and are not selectively enriched limiting total IL-2 produced and failing to accumulate enough IL-2 to overcome thresholds required for T cell activation.

However, the exact manner of by which the cytokine is produced is critical to its success. First, cytokine production must be dynamically regulated (inducible). Constant production of IL-2 risks exacerbating off-target toxicity. Moreover, constitutive IL-2 expression in T cells has negative effects – it leads to terminal differentiation, fails to drive autonomous proliferation, and is limited by payload silencing. Second, in order to bypass TCR/CAR suppression by the tumor microenvironment, induction of IL-2 production must be independent of the TCR activation pathway (e.g. NFAT promoter induced IL-2 still requires TCR/CAR activation to be triggered). We find that one powerful solution to this constraint is to engineer a synthetic signal transduction pathway that is tumor-triggered, but bypasses the native CAR/TCR activation pathway (Fig 6A,B). Using a synNotch receptor that detects the tumor to drive IL-2 production provides a simple and modular way to achieve this goal. The synNotch IL-2 circuit can maintain payload expression in spite of T cell inhibition or exhaustion (Fig S17A).

Finally, we find that simply having an immune cell that can individually produce high levels of IL-2 in the tumor is not sufficient to overcome suppression. The specific circuit architecture is critical, including exactly which cells produce IL-2. We find that an effective therapeutic response is only observed with an autocrine IL-2 circuit (i.e. synthetic IL-2 induction pathway is contained within the same cell as the anti-tumor CAR/TCR).

### Mechanisms underlying autocrine/paracrine circuit differences

Why does the autocrine IL-2 induction circuit perform so much better than the equivalent paracrine circuit in driving T cell infiltration of immunosuppressed tumor models? Both circuits act by the same principle of delivering high levels of IL-2 (Fig S17B) directly to the tumor. Moreover, why do we only see this large difference in autocrine vs paracrine circuit efficacy in the presence of a native immune system?

It is likely that there are multiple mechanisms that contribute to the far better efficacy of the autocrine circuit (Figure 6C,D). These mechanisms are tightly interlinked, and likely act in a highly cooperative manner, thus making it difficult to precisely pinpoint the relative contribution of each mechanism.

First, it is likely that autocrine cells have *preferential access* to self-produced IL-2, especially in environments with competing IL-2 sinks. Paracrine circuits must physically transfer IL-2 further through space from a producer T cell to an effector T cell. This becomes challenging in the presence of competing IL-2 consumer cells (e.g. T_regs_ in immune competent models), which can greatly reduce the effective length scale of IL-2 signaling creating gradients that drop off sharply around IL-2 sources (46). Here, in both the autocrine and paracrine circuit, we observe an expansion of host T_reg_ cells (Fig 5A, S15A); however, we also see a much larger expansion of naïve T cells (Fig S12B, S15A). These results suggest that host conventional T cells in the tumor can also play a significant role as IL-2 sinks, especially given their vast excess population. Although it is difficult to parse out the relative contribution of these T_regs_ vs conventional T cells as IL-2 consumers, it is not uncommon to observe the presence of large numbers of tumor infiltrating but non tumor-reactive T cells (30). Whatever their relative contribution, these IL-2 consumers are both expected to decrease the effective signaling distance of IL-2 producers (47, 48), which would strongly favor the efficacy of autocrine over paracrine IL-2 production in driving CAR T cell expansion.

Second, it also likely that autocrine cells are capable of *preferential expansion* in response to the available pool of IL-2. There is a unique proliferative positive feedback loop that could in principle take place with T cells that can induce both IL-2 and TCR/CAR activation. T cell activation can both trigger an initially IL-2 independent proliferative response (49) as well as induce expression of the high affinity IL-2 receptor subunit, CD25 (Fig S17C), which allows T cells to outcompete other T cells for available IL-2. Because an autocrine circuit cell contains both the CAR and synNotch→IL-2 circuit, it has the capability to become both a preferred IL-2 responder (via T cell activation) and strong IL-2 producer (via synNotch activation) within a tumor. We hypothesize that these dually activated autocrine cells could thereby initiate a powerful population level positive feedback loop that builds up even higher levels of intra-tumoral IL-2 due to preferential expansion of better IL-2 consumers/responders. This population positive feedback would not take place in the paracrine circuit, as the IL-2 producers do not upregulate CD25 (Fig S17C) and their IL-2 production would largely contribute to expanding competing T cells (such as Tregs) that act to suppress T cell based immunity. Several pieces of evidence support this model of preferred expansion of autocrine circuit cells. First, only in the autocrine CAR T cells, do we observe significantly higher expression of the proliferation marker Ki67 (Fig 5C, S14B). Second, we notably do not see increased tumor control with an autocrine circuit that produces the homeostatic cytokine IL-7 (Fig S17D). Further experiments will be needed to definitively evaluate the relative contributions of the multiple mechanisms discussed in this model.

### Essential requirements to bypass tumor immunosuppression

Our efforts to systematically design CAR T circuits that couple IL-2 production/signaling with CAR signaling in alternative ways also sheds light onto the basic design principles of native T cell activation. The T cell system has evolved to severely restrict improper activation, but at the same time to be able to launch a locally explosive response, once triggered. Population-level positive feedback signaling using a shared cytokine (IL-2) allows this type of digital response between on and off states (50, 51). In this model, T cells must not only be stimulated by the proper antigens, but they must also subsequently produce enough IL-2 to overcome the threshold set by competing IL-2 consumer cells present throughout the microenvironment (52). This control mechanism, however, provides weak points that tumors can take advantage of for immune suppression. Many tumors keep a strong T cell response in check, either by blocking T cell activation (28), or increasing competition for amplification factors like IL-2.

Here we show that it is possible to still reconstitute the pathways required for a strong anti-tumor T cells response (i.e. rewiring the cell such that T cell activation, co-stimulation and IL-2 signaling are still cooperatively stimulated), but in a way that now evades the major tumor suppressive mechanisms. Normally IL-2 is produced after T Cell activation and acts as a critical amplifier of T cell activity. By placing IL-2 production under the control of a new TCR-independent but still tumor-targeted synthetic receptor we can now produce IL-2 immediately and consistently after tumor entry despite suppression of T cell activation. In addition, normally IL-2 consumers apply a selective pressure only allowing strongly activated effector T cells to expand (52). By coupling TCR/CAR activation and synNotch driven IL-2 production in an autocrine IL-2 circuit we can selectively expand the engineered therapeutic T cell population out of a background of competing IL-2 consumers. These rewired cells ultimately activate the same critical pathways (TCR and IL-2 pathways) as seen in native T cell responses but do so in a different temporal order and in response to different inputs allowing them to be far more effective as a tumor-targeted therapy (Fig 6). The engineered circuit maintains the explosive cell expansion necessary for a robust anti-tumor activity but triggered in a manner that evades the major mechanisms of immunosuppression.

### The power of alternatively wired immune cell circuits.

In summary, we have been able to use flexible synthetic biology tools, such as the synNotch receptor system, to create new, alternative ways to rapidly establish both the TCR and IL-2 pathway activity required for an effective and sustained T cell response. The resulting bypass channel for IL-2 production allows for improved tumor control and reduced toxicity compared to alternative mechanisms of IL-2 delivery. Synthetic cytokine production circuits may represent a general solution for engineering immune cell therapies that can function more effectively in hostile tumor microenvironments, illustrating the power of customizing immune responses in highly precise but novel ways.

## Supplementary Material

Supp_Mats_Allen_GM

## Data Availability

All data are available in the main text or the supplementary materials.
