# Supplementary material for: Synthetic cytokine circuits that drive T cell infiltration into immune-excluded tumors
Source: Science. Author manuscript; Available in PMC 2023 Feb 27. (PMC9970000; doi:10.1126/science.aba1624)
Supplement: Supp_Mats_Allen_GM [file NIHMS1871154-supplement-Supp_Mats_Allen_GM.pdf]

Science
AAAS

Supplementary Materials for

Synthetic cytokine circuits that drive T cells into immune-excluded tumors

Greg M. Allen *et al*.

Corresponding author: Wendell A. Lim, wendell.lim@ucsf.edu

Science **378**, eaba1624 (2022)
DOI: 10.1126/science.aba1624

The PDF file includes:

Materials and Methods
Figs. S1 to S17
References

Other Supplementary Material for this manuscript includes the following:

MDAR Reproducibility Checklist

Materials and Methods

Viral DNA constructs

Primary human T cells were engineered with constructs cloned into a second generation 5' self-inactivating lentiviral backbone (pHR). All lentiviral constructs and sequences are detailed in Tables S1 and S2. Lentiviral synthetic cytokine circuits cells were made by transducing human T cells with a synNotch component and a response element component. SynNotch genes were expressed constitutively from mouse PGK promoters whereas response elements were controlled by a 5xGAL4 repeat with a minimal CMV promoter. The payloads controlled by synNotch were either expressed as "cytokine IRES mCherry" or "BFP P2A cytokine," where the cytokine was super-2 (sIL-2), human IL-2, IL-7, or IL-15. For in vitro experiments, bystander T cells were transduced with an SFFV eGFP vector. For in vivo experiments, the autocrine circuit was generated by placing the synNotch and response element in one lentiviral vector and either a SFFV effluc P2A mCherry or PGK BFP P2A anti-NY-ESO-1 TCR in the other vector. Paracrine circuits were created by placing the synNotch and response element in one cell and the effluc or NY-ESO-1 TCR in the other cell. Primary mouse T cells were engineered with constructs cloned into pMIG2 alternatively known as pMSCV or the self-inactivating pRetroX plasmid (Takara Bio). All retroviral constructs and sequences are detailed in Tables S3 – S6.

Primary immune cell culture

Primary human $CD8^+$ and $CD4^+$ T cells and NK cells were isolated from leukapheresis packs using EasySep kits (Stemcell Technologies), following which they were frozen in RPMI with 20% human AB serum and 10% DMSO. For assays, frozen T cells were thawed in human T cell media (hTCM; X-VIVO media [Lonza], 5% human AB serum, 10 mM n-acetyl cysteine, 55 μM β mercaptoethanol) with IL-2 (always 30 U/mL unless otherwise specified) and resuspended at 1e6 cells/mL. One day after thawing T cells were activated with 25 μL anti-CD3/anti-CD28 coated beads (Dynabeads Human T-Activator CD3/CD28 [Gibco]) per 1e6 T cells. 24 hours after activation T cells were infected by incubating 1e6 activated T cells with 1 to 1.5 mL of lentivirus for 24 hours. Following infection viral supernatant was removed from cells which were resuspended in media with IL-2. Cells were sorted for positive transduction on a FACSAria Fusion 5 days after activation based on expression of a fluorescent protein marker or for positive staining of a Myc-tag (anti-Myc-tag antibody, 9B11, Alexa Fluor 647 conjugate, Cell Signaling Technology, Cat# 2233) on synNotch, or both. Cells were then expanded by counting daily and diluting with hTCM with IL-2 to a cell concentration of 5e5 cells/mL for an additional week to allow cells to rest from initial activation prior to in vitro analysis or in vivo use. Frozen NK cells were thawed and resuspended in hTCM 24 hours prior to use.

Mouse T cells were isolated from spleens and lymph nodes of female C57/Bl6 or OT-1 mice, which were mechanically dissociated over a 40 micron filter. RBCs were lysed using RBC lysis buffer (Biolegend) prior to negative selection for $CD3^+$ or $CD8^+$ T cells (StemCell) with purity confirmed post-sort by surface staining. Mouse T cells were grown in RPMI supplemented with 10% fetal bovine serum, 2 mM Glutamax, 20 mM HEPES, 1% pen/strep, 1 mM sodium pyruvate, 0.05 mM beta-mercaptoethanol, and 50 IU/mL human IL-2. Mouse T cells were activated on day of isolation with either anti-mouse CD3/CD28 dynabeads (ThermoFisher) or OVA peptide (GenScript). 24 hours after activation 1e6 mouse T cells were spinfected at 2000g for 2 hours at 32C on retronectin coated (15 ug/mL, Takara Bio) non-TC coated 24 well plates with 1 to 1.5 mL of retrovirus and 4 ng/mL polybrene (Sigma-Aldrich). Retrovirus was removed

after 4 hours and mouse T cells expanded until 3 days after activation when they were sorted on a FACSaria as above. Mouse T cells were then expanded daily by counting and diluting with mTCM + IL-2 to maintain a concentration of 1e6 cells/mL for 9 days after activation prior to use with in vitro or in vivo assays.

Virus Production
Lentivirus was produced using Lx293t lentiviral packaging cells (Takara bio, Cat# 632180) that were seeded in 6-well plates at 7e5 cells/well and 24 hours later transfected with pHR constructs and pCMV and pMD2.g packaging plasmids using FuGene HD (Promega) following manufacturer's protocol. 48 hours after transfection viral supernatant was collected and filtered prior to use with human T cell cultures. Retrovirus was producing using Plat-E retroviral packaging cells (Cell Biolabs, Cat# RV-101) that were seeded in 6 well plates at 9e5 cells/well and 24 hours later transfected with pMIG2 or pRetroX constructs using FuGene HD (Promega) following manufacturer's protocol. 48 hours after transfection viral supernatant was collected and filtered prior to use with mouse T cell cultures.

Tumor Cell Culture
Human K562 cells were purchased from the ATCC (CCL-243) and were cultured in Iscove Modified Dulbecco's Modified Eagle Medium with 10% FBS and split to 2.5e5 cells/mL every 3 days or 3.5e5 cells/mL every 2 days. Human A375 cells were purchased from the ATCC (CRL-1619) and cultured in DMEM with 10% FBS and split 1:6 every 2 days or 1:10 every 3 days. K562 lines were transduced to constitutively expressed mCherry and/or CD19 ligand (membrane-tethered CD19 extracellular domain). A375 lines endogenously present NY-ESO-1 antigen and were transduced to express GFP ligand (membrane-tethered GFP), or not. Murine C57/Bl6 KPC (KrasLSL.G12D/+; p53R172H/+; PdxCretg/+) cells were a kind gift of the Stanger Lab (*25)* and were cultured in Dulbecco's Modified Eagle Medium with 10% FBS and split 1:5 every 2 days or 1:10 every 3 days. Murine C57/Bl6 B16F10 OVA cells were a kind gift of the Krummel Lab and were cultured in Dulbecco's Modified Eagle Medium with 10% FBS and split 1:5 every 2 days or 1:10 every 3 days.

In-Vitro Assays
T cells and target cells were washed of residual media and cytokines by two rounds of centrifugations at 400xg for 4 minutes followed by resuspension in hTCM without IL-2. In some cases, T cells were stained with 1:5000 CellTrace CFSE proliferation stain (Molecular Probes) following manufacturer's protocol. Magnetic anti-HA or anti-Myc-tag antibody-coated beads (Pierce) were washed 3 times with hTCM using a magnet before using. Immune cells were seeded at 2.5e5 cells/mL and K562 cells at 1.25e5 cells/ml. IL-2 was added as indicated. For beads, 2.5 μL (in terms of original suspension before washing) per well was used. Every 2 days, wells were triturated and 50 μL cell suspension was taken to analyze by flow cytometry. A 96-well magnet array was used to retain magnetic beads. The media was then replenished: cells were pelleted, 100 μL of old media was removed, then 150 μL fresh hTCM was added to restore the volume to 200 μL.

Mouse Experiments
Prior to injection into mice, T cells were washed in PBS, resuspended at 10 times the injection amount per mL, and 100 μL was injected via the tail vein on day 0. Mouse T cells were

administered to female 6 to 12 week old C57/Bl6 mice (Jackson Labs Strain #000664) and human T cells were administered to female 6 to 12 week old NSG (NOD-*scid* IL2Rgamma^{null}) mice. Heterotopic K562, A375 and KPC tumors were prepared by washing cells in PBS three times, resuspending at 10 times the injection amount per mL, and injecting 100 µL subcutaneously in each flank on day 0 (K562 tumors), day –4 (A375 tumors) or day -7 to -9 (KPC tumors). Orthotopic B16-F10 tumors were prepared as before but resuspended at 25 times the injection amount per mL, and 25 uL was injected intradermally on day -7 to -9. Orthotopic KPC tumors were prepared as before with 125,000 cells implanted into the tail of the pancreas in 25 uL in a 1:1 mix with Matrigel (Fisher Scientific). Sub-cutaneous and intra-dermal tumor size was measured by caliper while orthotopic tumor size was measured by luciferase signal. Bioluminescence imaging was performed using an IVIS Spectrum (Perkin Elmer). Mice were injected intraperitoneally with 200 µL of 15 mg/mL d-luciferin (Goldbio) and imaged 15 minutes later. Typical sample size per group was 5 mice with randomization at time of tumor implantation. Tumor measurements were performed by staff blinded to treatment groups, only mice that were engrafted tumors were included in analysis. All animal studies were performed under the UCSF Office of Research Institutional Animal Care and Use Program approval number AN183960-02N.

In-Vitro Immune Cell Assays

Flow cytometry was performed either on a BD LSRii or BD Fortessa X-20 with high-throughput system. Analysis of flow data was performed in FlowJo (FlowJo, LLC). For counting, constant volumes were taken from each well and all events were counted within those volumes. IL-2 production in primary human T cells was measured in 96 well flat bottom plates with A375 cells in co-culture with T cells. After 24 hours T cells were exposed to golgi-plug/stop (BD Biosciences) for four hours and then fixed and stained with PE-Texas Red anti-CD4 (Biolegened, Cat# 317448, AB_2565847) and BUV-737 conjugated anti-human IL-2 (BD Bioscience, Cat# 564446) before flowing. ELISA measurements of IL-2 production from mouse T cells were performed using a human or mouse IL-2 quantikine ELISA kit (R&D systems, Cat# M2000). Absolute T Cell proliferation was measured by adding 2 uL of anti-myc Tag beads to T cells in a 200 uL volume on Day 0 and sampling 50 uL of cells with replacement of media every 2 days. Viable T Cell counts were quantified using 1:500 Sytox (Molecular Probes) in PBS and accounting for dilution of wells. T cell proliferation was also analyzed by dilution of CFSE cell trace dye (Thermo Fisher). NK cell expansion was measured in co-culture of T cells, NK cells and K562 cells. Cells were stained with 1:100 anti-CD3 and 1:100 anti-CD8 fluorescent conjugated antibodies in flow buffer (PBS, 5% FBS) for 30 minutes at 4°C followed by 1:500 DRAQ7 (Molecular Probes) in flow buffer. Mouse T cell cytotoxicity was tested against GFP labeled target cells that were plated at indicated count in 100 uL in 96 well flat bottom plates and placed in an Incucyte Live-Cell Imaging System (Sartorius). T cells were added at indicated count in 100 uL of mTCM without IL2 and survival of GFP labeled target cells was recorded by imaging. Mouse T cell differentiation measured with BV605 anti-PD-1 (Biolegend, Cat# 135220, AB_2562616) and PE Lag3 (Biolegend, Cat #125208, AB_2133343). Human T cell differentiation measured with FITC anti-CD450RO (Biolegend, Cat #304242, AB_314420) and AF647 anti-CCR7 (Biolegend, Cat #353217, AB_10913812). Cytokine receptor expression measured with BV-421 anti-IL7R (Biolegend, Cat# 351309, AB_10898326), APC anti-IL15Ra (Biolegend, Cat #330209, AB_2561439), PE-TR anti-CD25 (Biolegend, Cat # 302645, AB_2734259).

Analysis of tumor specimens: IHC

Tumors samples were collected for IHC and fixed in 10% formalin for 24 to 48 hours prior to preservation in 70% ETOH. Tissue was embedded in paraffin, sectioned and mounted for staining with single chromogenic anti-CD3 (clone SP7) antibody at the UCSF Biorepository core facility.

Analysis of tumor specimens: Flow cytometry

Tumor samples for flow cytometry were collected and immediately processed. Tumors were finely minced, digested in a mixture of 1 mg/mL collagenase IV, 20 U/mL DNAse IV, and 0.1 mg/mL hyaluronidase V for 30 min at 37°C with shaking, passed through 70 µm cell strainers, washed twice with PBS + 0.04% EDTA, and aliquoted in 96 well plates for high-throughput flow cytometry. Cells were stained with 1:500 Live/Dead (Molecular Probes) in flow buffer for 15 minutes, followed by additional surface staining. Antibodies used to profile murine tumors included an AF700 anti-CD45 (Biolegend, Cat# 103127, AB_493714), PE-594 anti-CD3 (Biolegend, Cat# 100245, AB_2565882), APC anti-CD4 (Biolegend, Cat# 100411, AB_312696), BV711 anti-CD8 (Biolegend, Cat# 100747, AB_11219594), and APC-Cy7 anti-Thy1.1 (Biolegend, Cat# 202519, AB_2201418). Flow cytometry was performed on a BD Fortessa X-20 with high-throughput system and analysis of flow data was performed in FlowJo (FlowJo, LLC), compensation was performed with Ultracomp eBeads (ThermoFisher).

Analysis of tumor specimens: CyTOF (Cytometry by time of flight)

Tumor samples for CyTOF were processed immediately after collection. Tumor samples were minced and digested in RPMI 1640 with 1 mg/ml collagenase IV and 0.1 mg/ml DNase I for 30 min at 37°C with shaking. Digested tumor samples were filtered with a 70 µm cell strainer and washed with PBS + 5mM EDTA at 4°C. Cells were resuspended 1:1 with PBS + 5mM EDTA + 50µM Cisplatin (Sigma) for exactly 60s prior to quenching 1:1 with PBS + 5mM EDTA + 0.5% BSA to determine viability. Tumor sample was fixed for 10 min at room temperature using 1.6% PFA and frozen at -80°C. Mass-tag cellular barcoding and antibody staining of samples for CyTOF were performed as previously described (*1*). A summary of all antibodies used for CyTOF are detailed in Tables S7. All antibodies were conjugated at the UCSF Parnassus Flow Cytometry Core. Each antibody clone and lot was titrated to optimal staining concentration using primary murine samples. CyTOF was performed on a Helios mass cytometer (Fluidigm) with data analysis performed in CellEngine and R.

SUPPLEMENTAL FIGURES

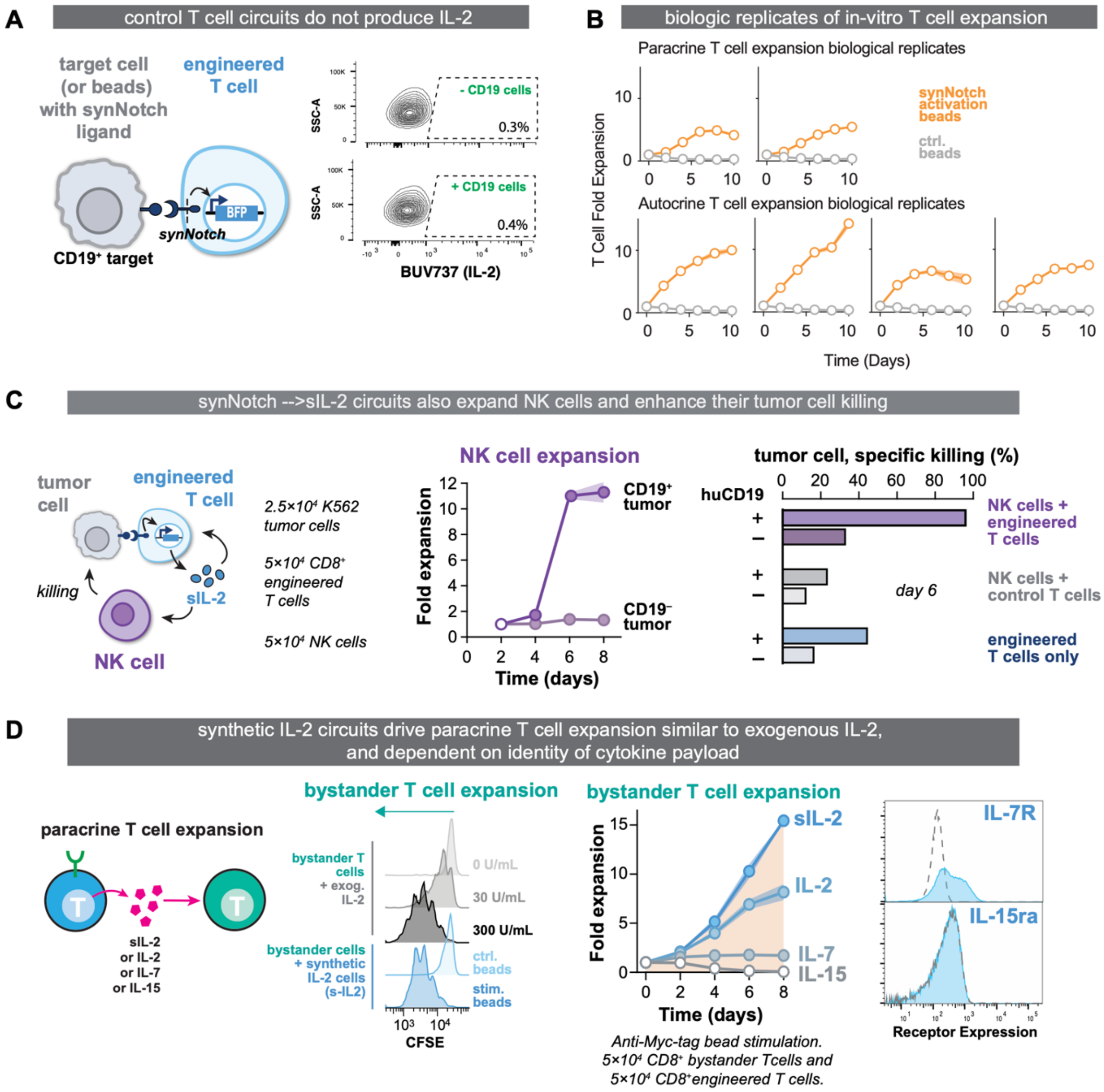

Fig. S1. Driving immune cell expansion with synNotch=>IL-2 circuits.
(**A**) Control Human T cells engineered with an anti-CD19 synNotch driving production of a *dummy* payload (blue flourescent protein) do not produce IL-2 (as measured by intracellular flow) when co-cultured with CD19+ or CD19- A375 tumor cells, compare to **Figure 1B**.
(**B**) Additional biologic replicates of human T cell expansion when engineered with an anti-CD19 SynNotch => sIL-2 circuit in paracrine (top) or autocrine (bottom) from different donor, compare to **Figure 1C and 1D**.
(**C**) (*left*) Synthetic IL-2 circuit T cells stimulate expansion and cytotoxicity of NK cells in a paracrine fashion. (*middle*) tumor antigen-specific NK cell expansion by synthetic IL-2 circuit T cells. (*right*) Specific killing of tumor cells by NK cells when co-cultured with synthetic IL-2 circuit (engineered) T cells is antigen-specific and exceeds killing with control T cells or synthetic IL-2 circuit (engineered) cells alone.
(**D**) Synthetic IL-2 circuit T cells delivering different cytokines to expand bystander T cells. (*center*) Shown here is bystander cell CFSE dye proliferation assay; cell division creates peaks of successively lower fluorescence. Bystander cells co-cultured with synthetic IL-2 circuit cells and anti-Myc-tag beads (blue histograms) proliferated similarly to those treated with high doses of exogenous IL-2 (grey histograms). (*right*) Measurements of bystander T cell proliferation when synthetic synNotch=>IL-2 circuit T cells producing labeled cytokine were activated. Filled markers: significant expansion>1, right-tailed Students t-test, $p<0.05$. Error shading in: S.E.M. (*far right*) Expression of IL-7R and IL-15R alpha in primary human CD8+ T cells used in proliferation assay. Dash grey line indicates isotype control, filled blue line cell staining.

Supplemental Figure 2 (assoc with Figure 1)

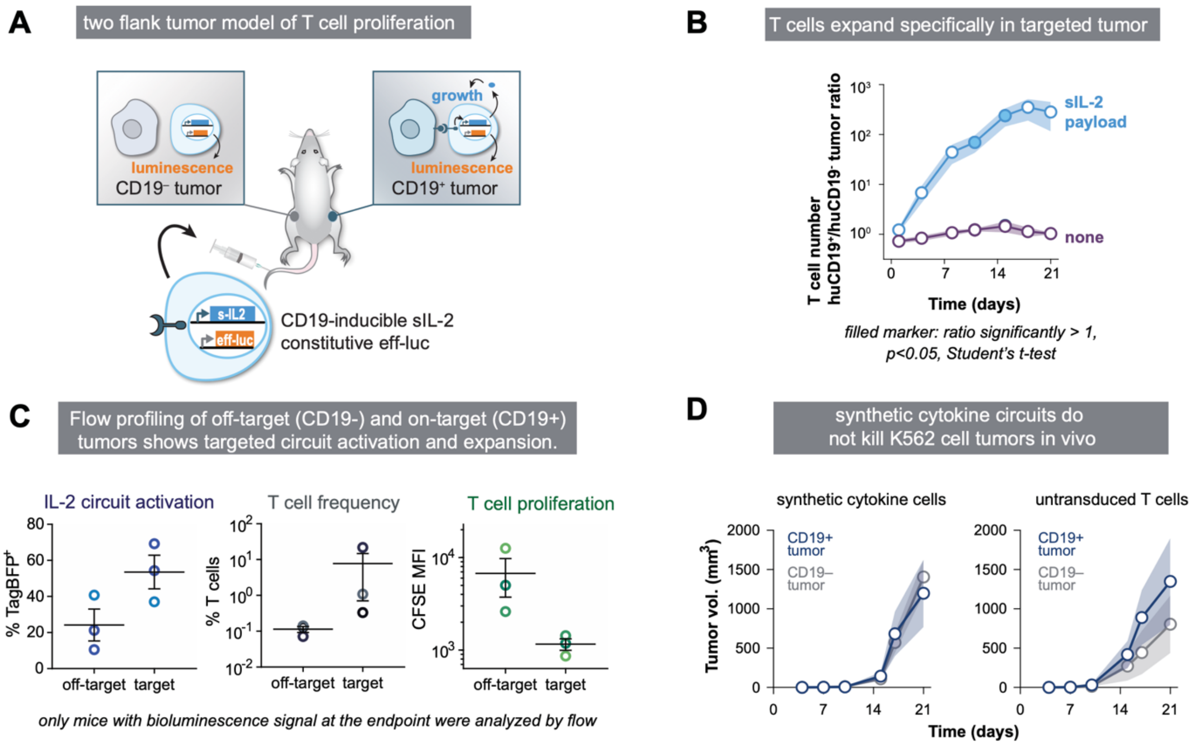

Fig. S2. Local autocrine proliferation *in vivo* driven by synthetic IL-2 circuit

(**A**) Bilateral tumor model for local proliferation as shown in **Figure 1**. Right and left flank tumors: CD19+ and CD19– K562 cells, respectively. Human T cells expressing anti-CD19 synNotch driving sIL-2 and constitutively expressing eff-luc were injected intravenously and T cell localization monitored by serial bioluminescence.
(**B**) Ratio of bioluminescence signal from on-target tumor (CD19+) to off-target tumor (CD19-) shows selective T cell expansion in the targeted CD19+ tumor for cells engineered with a cytokine circuit producing sIL-2 (blue) but not in cells that don't express a cytokine payload (violet). Filled markers: significant ratio>1, right-tailed Student's t-test, $p<0.05$.
(**C**) In the CD19+ compared to CD19– tumor, T cells were more likely to have the synthetic IL-2 circuit activated as measured by co-produced BFP marker (*left*) and were more frequent as a proportion of live cells in the tumor (*middle*) with evidence for more T cell proliferation as measured by dilution of CFSE membrane dye that T cells were stained with prior to injection (*right*).
(**D**) Tumor volume of mice given CD19 targeted synthetic IL-2 circuit expressing T cells (left) or untransduced T cells (right), comparing $CD19^+$ (dark blue) to $CD19^-$ (gray) tumors. The synthetic IL-2 circuit alone in NSG mice had no effect on tumor growth. Error shading: S.E.M. Open markers indicate no significant difference between tumors, Student's t-test, $p>0.05$.

Supplemental Figure 3 (assoc with Figure 1)

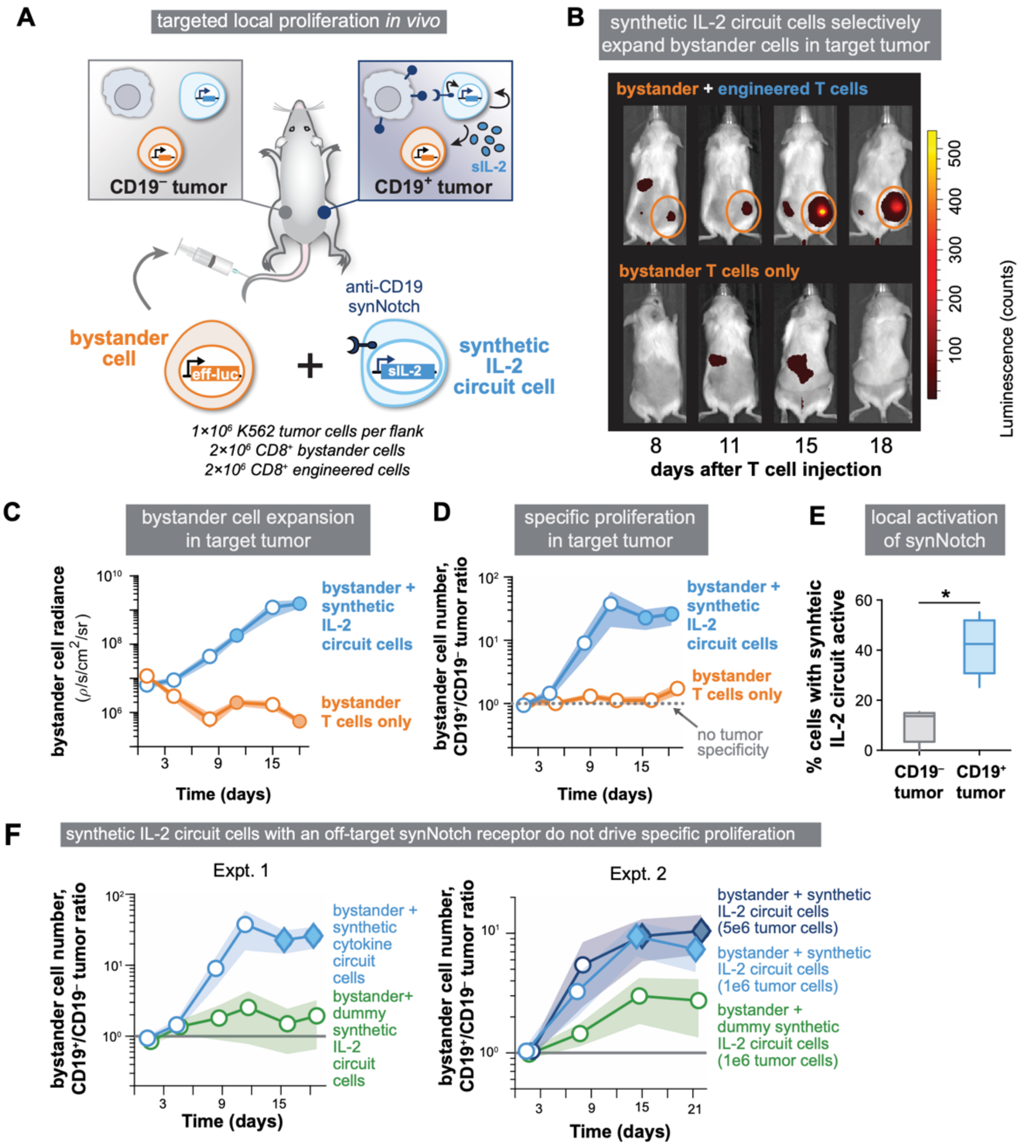

Fig. S3. Local paracrine proliferation *in vivo* driven by synthetic IL-2 circuit.
(**A**) Bilateral tumor model for local proliferation. Right and left flank tumors: CD19$^+$ and CD19$^-$ K562 cells, respectively. Bystander T cells expressing eff-luc were co-injected with synthetic IL-2 circuit T cells (anti-CD19 synNotch => sIL-2).
(**B**) Bioluminescence imaging of eff-luc-expressing bystander T cells. Circles highlight proliferation in the CD19$^+$ tumor in mice that also recieved synthetic IL-2 circuit T cells. One mouse per group is shown.
(**C**) Bystander cell radiance in CD19$^+$ tumor rose in mice receiving both cell types (blue), but fell with bystander cells only (orange). Filled marker: significant difference between groups, Student's t-test, $p<0.05$. Error shading in C, D: S.E.M.
(**D**) Tumor specificity: ratio of bystander cell radiance in CD19$^+$ to CD19$^-$ tumor. Bystander cell proliferation was specific for the CD19$^+$ tumor only with co-injection of CD19 targeted synthetic IL-2 circuit cells. (Filled markers: significant ratio>1, right-tailed Student's t-test, $p<0.05$.
(**E**) The Synthetic IL-2 circuit was only activated in the targeted (CD19$^+$) tumor. Activation measured by expression of mCherry (co-expressed with s-IL2). * indicates significant difference by Student's t-test, $p<0.05$, and boxplot shows, median, quartiles, and extent of data.
(**F**) Antigen specificity of bystander cell proliferation, determined by the ratio of bystander cell bioluminescence in the CD19$^+$ to CD19$^-$ tumor. Comparison between groups of mice given bystander cells with synthetic IL-2 circuit cells (blue or dark blue for 1e6 or 5e6 tumor cells implanted) or "dummy" synthetic IL-2 circuit expressing a synNotch for an irrelevant antigen (anti-mGFP; green), across two experimental replicates (left and right). Error shading: S.E.M. Filled markers: ratio>1, right-tailed Student's t-test, $p<0.05$.

Supplemental Figure 4 (assoc with Figure 1)

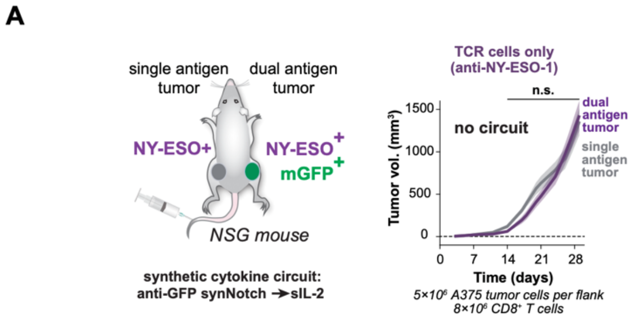

Individual growth curves for tumor volumes in Figure 2.

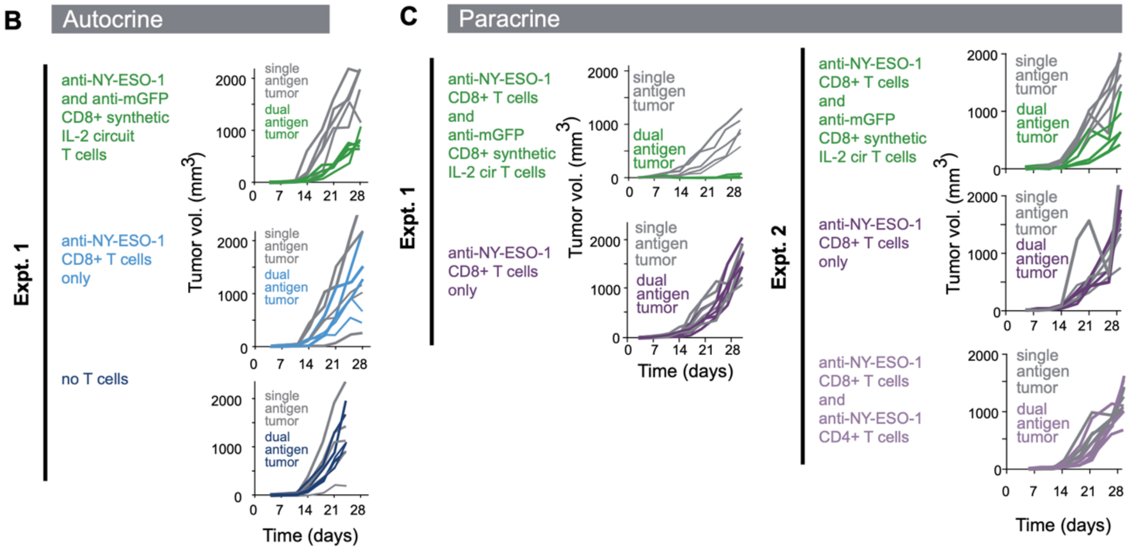

Fig. S4. Tumor killing enhanced by synthetic IL-2 circuits in individual mice.
(**A**) Two-flank A375 tumor model in NSG mice, with NY-ESO only on left and NY-ESO/GFP on right. Plots show tumor growth over time. T cells with only anti-NY-ESO TCR do not clear either tumor. Error shading: S.E.M.
(B) Individual mouse tumor volume trajectories given anti-NY-ESO-1 TCR CD8+ T cells engineered with a synthetic IL-2 circuit (*green*), anti-NY-ESO-1 TCR only CD8+ T cells (*light blue*), or no T cells (*dark blue*).
(C) Individual mouse tumor volume trajectories given anti-NY-ESO-1 TCR cells with (*green*) or without (*violet*) synthetic cytokine cells, or with anti-NY-ESO-1 CD4+ T cells (*light violet*) instead of synthetic IL-2 cells, across two experimental replicates. For A,B: dual antigen tumor (*colored line*) compared to single antigen tumor (*gray line*) volume.

Supplemental Figure 5 (assoc with Figure 1)

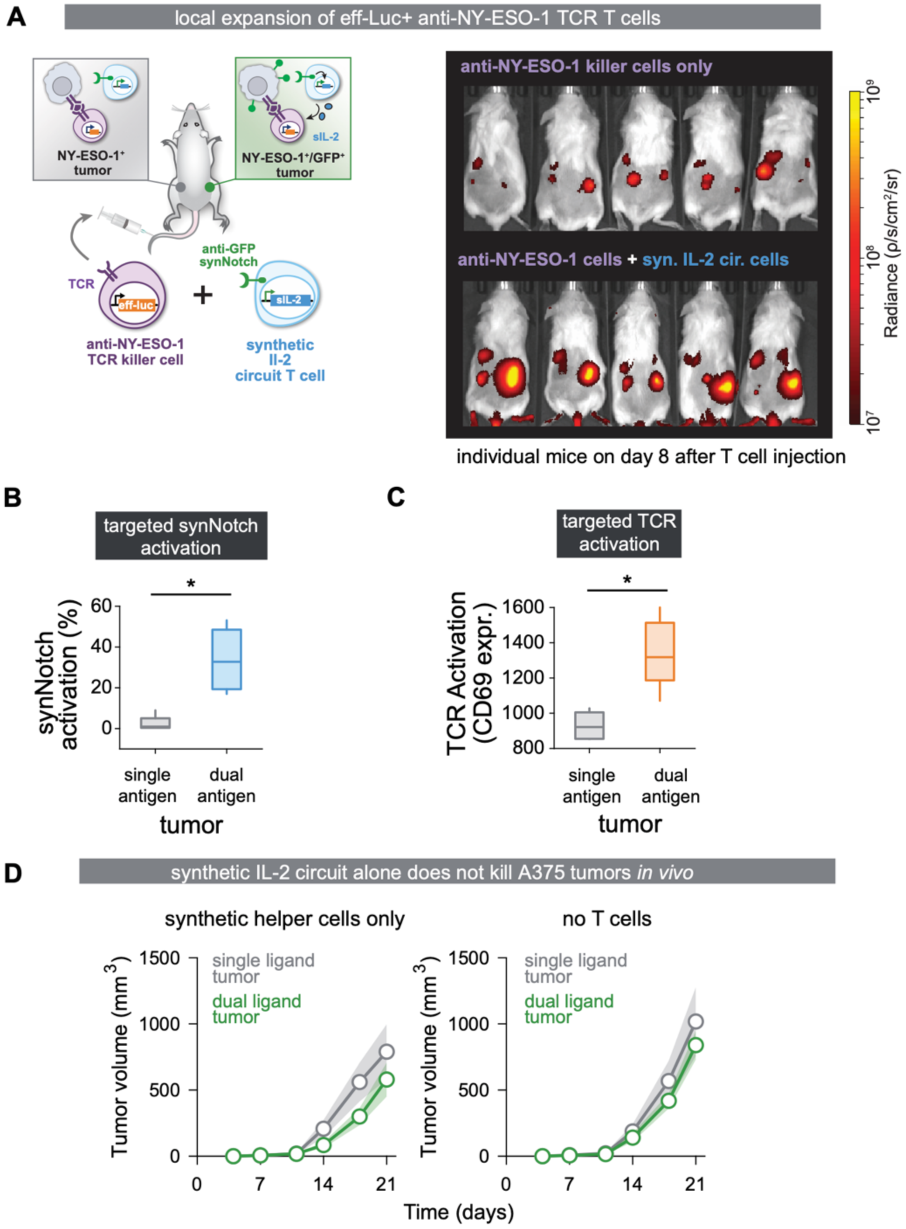

Fig. S5. Enhanced local proliferation of tumor-reactive TCR T cells when co-delivered with synthetic IL-2 circuit T cells.
(**A**) Bilateral tumor model to test local killing enhancement by synthetic IL-2 circuit cells. Left tumor (single antigen): A375 malignant melanoma cells natively presenting NY-ESO-1 antigen. Right tumor (dual antigen): mGFP+ A375 cells. Killer T cells expressing anti-NY-ESO-1 TCR and eff-luc were co-injected with synthetic IL-2 circuit T cells expressing anti-mGFP synNotch driving sIL-2. Bioluminescence of killer cells at day 8 shows specific proliferation in the dual antigen tumor only with synthetic IL-2 circuit T cells.
(**B**) Flow cytometry analysis of treated tumors show specific activation of the synthetic IL-2 circuit only in the dual antigen tumor. Activation measured by expression of mCherry (co-expressed with sIL-2).
(**C**) Flow cytometry analysis of treated tumors show increased markers of T cell activation (CD69) only in the dual antigen tumor. In E,F: difference between dual antigen/single antigen tumors by Student's t-test, $p<0.05$. Boxplot shows, median, quartiles, and extent of data.
(**D**) Tumor volume of mice given synthetic IL-2 circuit cells only (left) or no T cells (right), comparing dual (*green*) and single (*gray*) antigen tumors. Error shading: S.E.M. Open markers represent no significant difference between tumors, Student's t-test, $p>0.05$.

Supplemental Figure 6 (assoc with Figure 2)

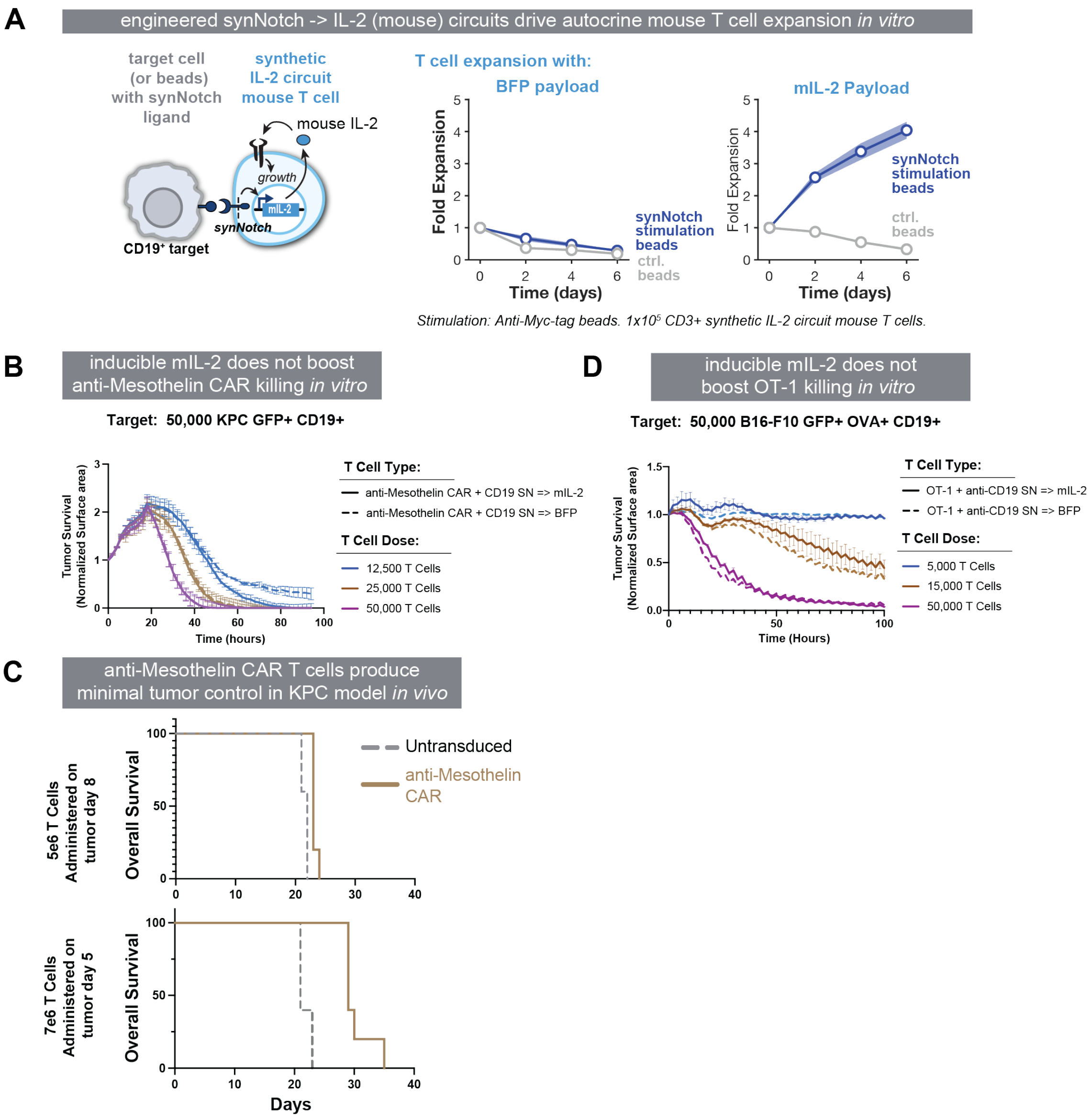

Fig. S6. In-vitro characterization of engineered mouse T cells used in syngeneic tumor model experiments.
(**A**) Primary mouse CD3⁺ T cells were engineered with a single retroviral vector encoding an inducible BFP or an inducible mIL-2 payload driven by an anti-CD19 SynNotch. After resting from initial CD3/CD28 expansion, IL-2 was removed from the media and synNotch receptor activated using anti-Myc beads (receptor is myc tagged). T cells were seen to proliferate specifically with synNotch activation only.
(**B**) Primary mouse mouse CD3⁺ T cells were engineered with an anti-Mesothelin CAR T and either an anti-CD19 SynNotch inducible BFP or anti-CD19 SynNotch inducible mIL-2. The indicated number of T cells were incubated with 50,000 GFP⁺ KPC CD19⁺ target cells and tumor cell survival was measured in real time with an Incucyte Live Cell Imaging system. There was no apparent difference in tumor survival when tumor cells were co-cultured with cytotoxic T cells engineered to make mIL-2.
(**C**) Survival of mice after eff-luc⁺ CD19⁺ KPC tumors were implanted in the tail of the pancreas and treated with labeled number of sorted anti-Mesothelin CD3⁺ CAR T cells on the indicated number of days after tumor implantation. CAR treatment alone provided minimal tumor control even at high T cell doses given at early time points with no mice surviving.
(**D**) As in (*B*) cytotoxicity of mouse OT-1 CD8⁺ T cells engineered to express either an anti-CD19 synNotch inducible BFP or anti-CD19 synNotch inducilbe mIL-2 targeting 50,000 B16-F10 GFP+ OVA+ CD19+ tumor cells.

Supplemental Figure 7 (assoc with Figure 2)

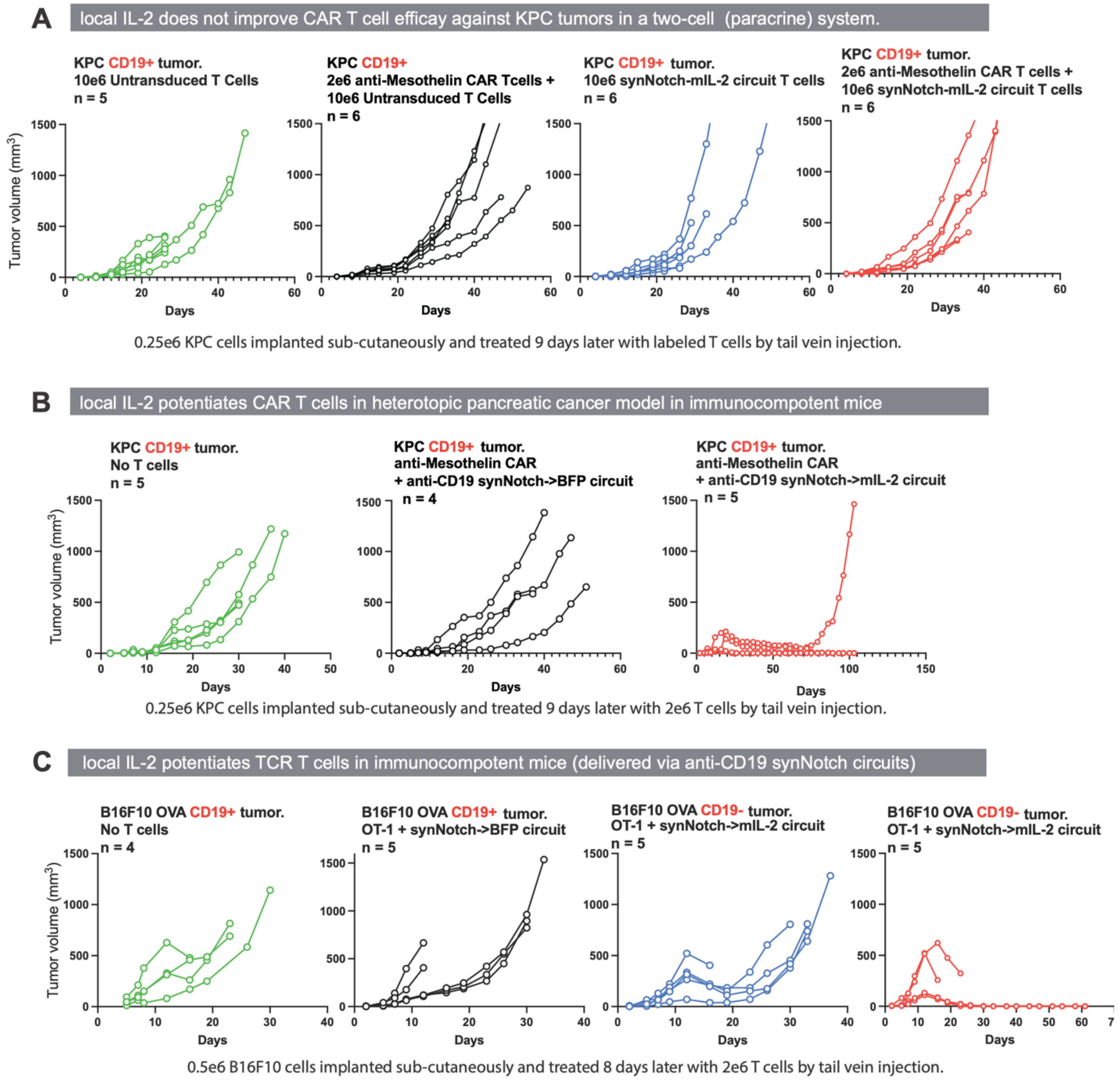

Fig. S7. Individual mouse tumor volume data for experiments showing that syngeneic tumor killing is enhanced by synthetic IL-2 circuits (synNotch=>mIL-2):

Plots of individual growth curves from tumors in specified mice.
(**A**) 250,000 sub-cutaneously implanted KPC CD19^{+} tumor cells treated with T cell doses as labeled, matching Figure 3B.
(**B**) 250,000 sub-cutaneously implanted KPC CD19^{+} tumors cells treated with 2e6 mouse T cells engineered as labeled, matching Figure 3C.
(**C**) 500,000 sub-cutaneously implanted B16F10 OVA tumor cells (+/- CD19 as labeled) treated with 2e6 mouse T cells engineered as labeled, matching Figure 3E.

Supplemental Figure 8 (assoc with Figure 2)

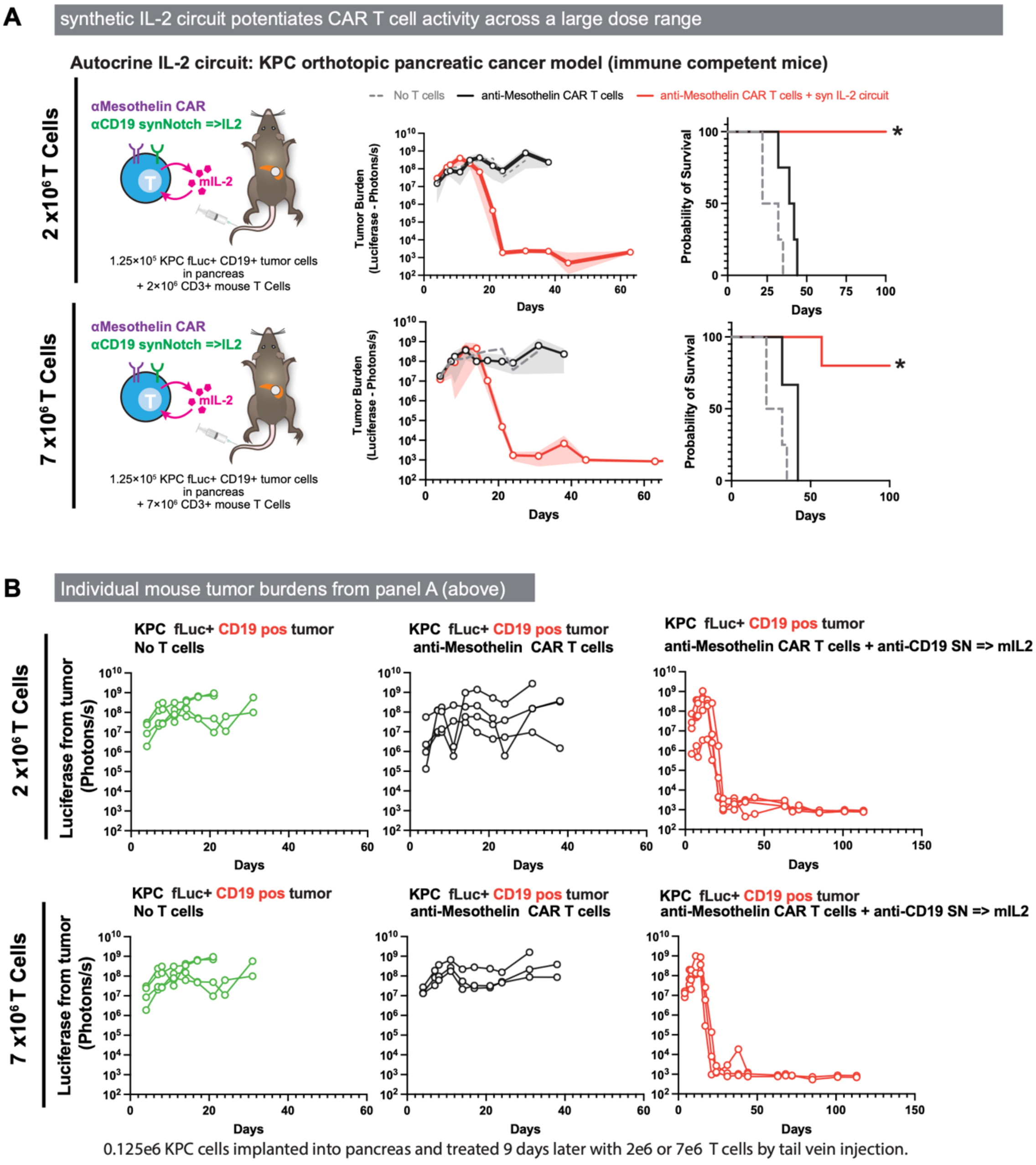

Fig. S8 Orthotopic syngeneic tumor killing enhanced by synthetic cytokine circuits
(A) KPC $CD19^+$ $fLuc^+$ pancreatic tumors were engrafted orthotopically into tail of the pancreas into immunocompotent C57/Bl6 mice and treated 9 days later with 2e6 or 7e6 engineered mouse $CD3^+$ T cells by tail vein injection. Tumor control was only seen with anti-Mesothelin CAR T cells engineered with a synthetic IL-2 circuit. Plots show tumor burden as measured by average +/- S.E.M. of luciferase signal from abdominal cavity and overall survival (n=4-5 per group, * = significant difference in survival with addition of IL-2 circuit using log-rank test, $p < 0.05$).
(B) Plots of individual growth curves from tumors in specified mice in panel A.

Supplemental Figure 9 (assoc with Figure 3)

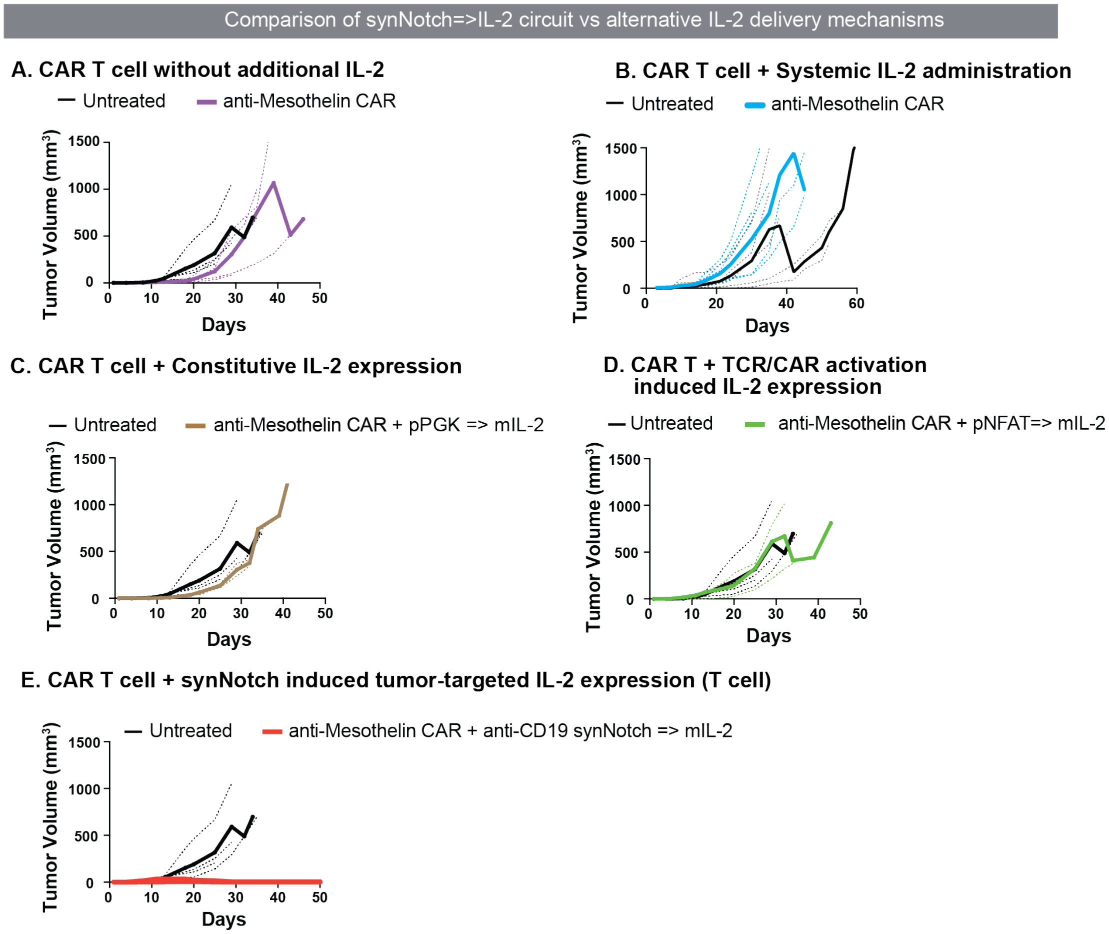

Fig. S9. Comparison of alternative strategies to deliver IL-2 to enhance T cell cytotoxicity against tumors.
Tumor growth curves corresponding to Figure 3. KPC CD19+ pancreatic tumors were engrafted subcutaneously into immunocompetent C57/Bl6 mice and treated 9 days later with T cells as labeled. Plotted is individual (dash line) and mean (solid line) growth curves for each cell design compared to matched untreated mice. n=4,5 per group.
(A) 1e6 anti-Mesothelin CAR T cells with no additional IL-2.
(B) 2e6 anti-Mesothelin CAR T cells with systemic IL-2 administered at high dose (250,000 to 750,000 IU/mL) twice daily intraperitoneally for 7 days.
(C) 1e6 anti-Mesothelin CAR T cells engineered to constitutively express mIL-2 using a PGK promoter.
(D) 1e6 anti-Mesothelin CAR T cells engineered to inducibly express mIL-2 under the control of a NFAT promoter.
(E) 1e6 anti-Mesothelin CAR T cells engineered to inducibly express mIL-2 under the control of an anti-CD19 synNotch.

Supplemental Figure 10: assoc with Figure 3

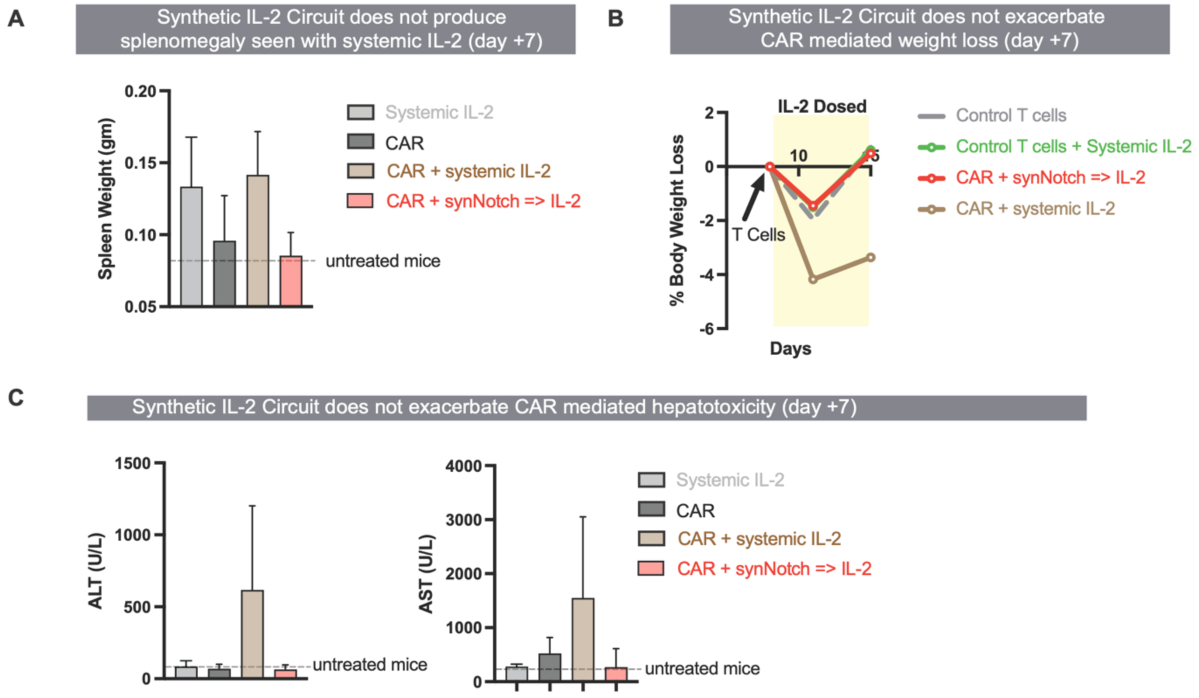

Fig. S10. Evaluating toxicity: SynNotch-->IL-2 circuit does not produce systemic toxicity or worsen CAR toxicities.
To assess potential toxicity of a synthetic IL-2 circuit, C57/Bl6 mice were engrafted with KPC CD19^{+} tumors sub-cutaneously and given either 2e6 control untransduced T cells +/- systemic IL-2, 2e6 anti-Mesothelin CAR T cells +/- IL-2, or 2e6 anti-Mesothelin CAR T cells with an anti-CD19 SynNotch => IL-2 circuit. Systemic human IL-2 was given at high dose (250,000 to 750,000 IU/mL) twice daily intraperitoneally for 7 days. 7 days after T cell treatment, 3-5 mice per group were sacked for analysis of toxicity measurements and the remaining mice were monitored for survival.

(**A**) Systemic IL-2 caused splenomegaly in all mice, but no splenomegaly was seen with the synthetic cytokine circuit. This IL-2 mediated toxicity was seen in presence or absence of anti-Mesothelin CAR T cells.
(**B**) Systemic IL-2 combined with the anti-mesothelin CAR also induced a more profound weight loss in mice when compared with CAR T cells combined with a synthetic IL-2 circuit or systemic IL-2 only, again suggesting that systemic IL-2 can exacerbate CAR T cell toxicities.
(**C**) Systemic IL-2 combined with CAR T cell therapy produced significant hepatotoxicity (as measured by ALT and AST) in mice that recieved CAR and systemic cytokine. No similar toxicity was seen with systemic IL-2 only or when CAR was combined with a synthetic IL-2 circuit. This suggests that sytemic IL-2 can exacerbate CAR T cell toxicities, which may include on-tumor/off-target CAR t cell reactions against normal tissue (liver).

Supplemental Figure 11: assoc with Figure 3

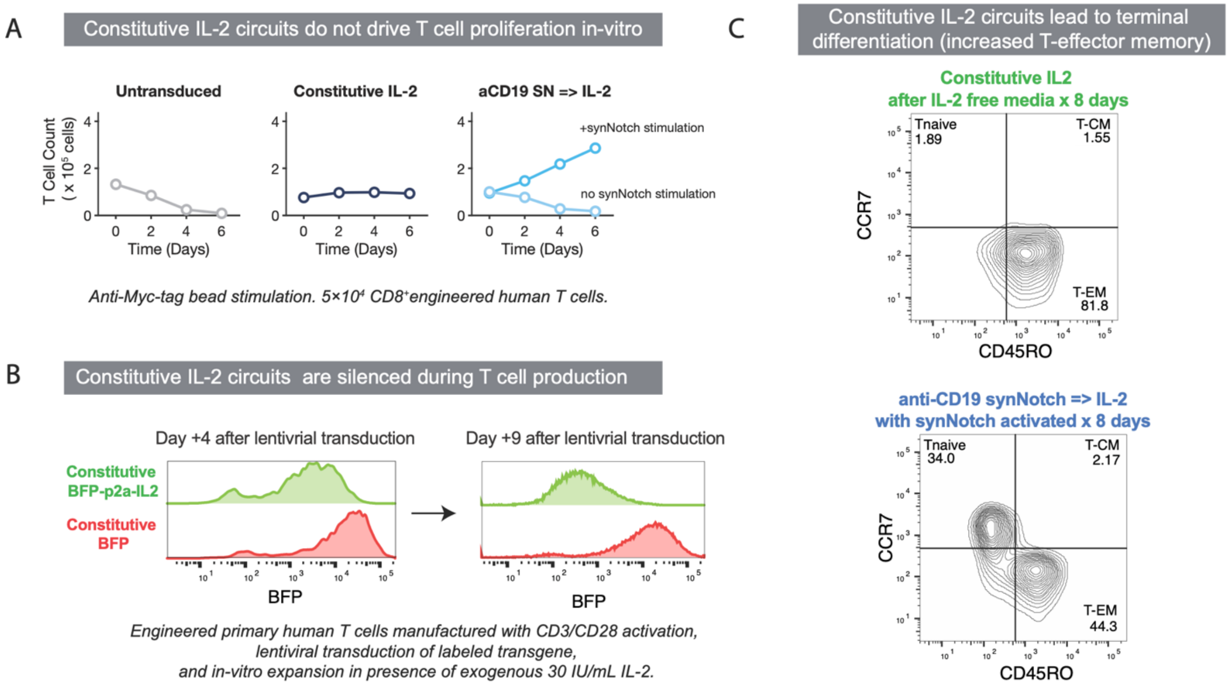

Fig. S11. Comparison of effect of constitutive vs inducible IL-2 on T cell behavior.
(**A**) Primary human T cells that were either unmodified, engineered to express a constitutive sIL-2 payload (PGK promoter) or an anti-CD19 synNotch inducible sIL-2 were assesed for their ability to autonomously proliferate in IL-2 free media as in Figure 1. Constitutive IL_2 production led to cell survival but not expansion, when compared to synNotch inducible IL-2 production.
(**B**) sIL-2 expression from human T cells was approximated by use of co-expressed BFP marker (using a 2a element with a PGK promoter). During 5 days of in-vitro culture significant silencing of BFP marker was seen for the BFP-p2a-IL2 plasmid but not for a BFP only plasmid expressed in matched T cells, suggesting constitutive IL-2 circuits are selected against.
(**C**) Primary human T cell differentiation was approximated using surface marker staining for CCR7 and CD45RO. Constitutive IL-2 production led to almost all cells being in a CD45RO+CCR7- effector memory state. In comparison matched T cells expressing an anti-CD19 synNotch => IL-2 circuit that was triggered using anti-myc beads (synNotch receptor is myc tagged) show far less terminal differentiation and maintenance of a CD45RO-CCR7+ naive popultion.

Supplemental Figure 12 (assoc with Figure 4)

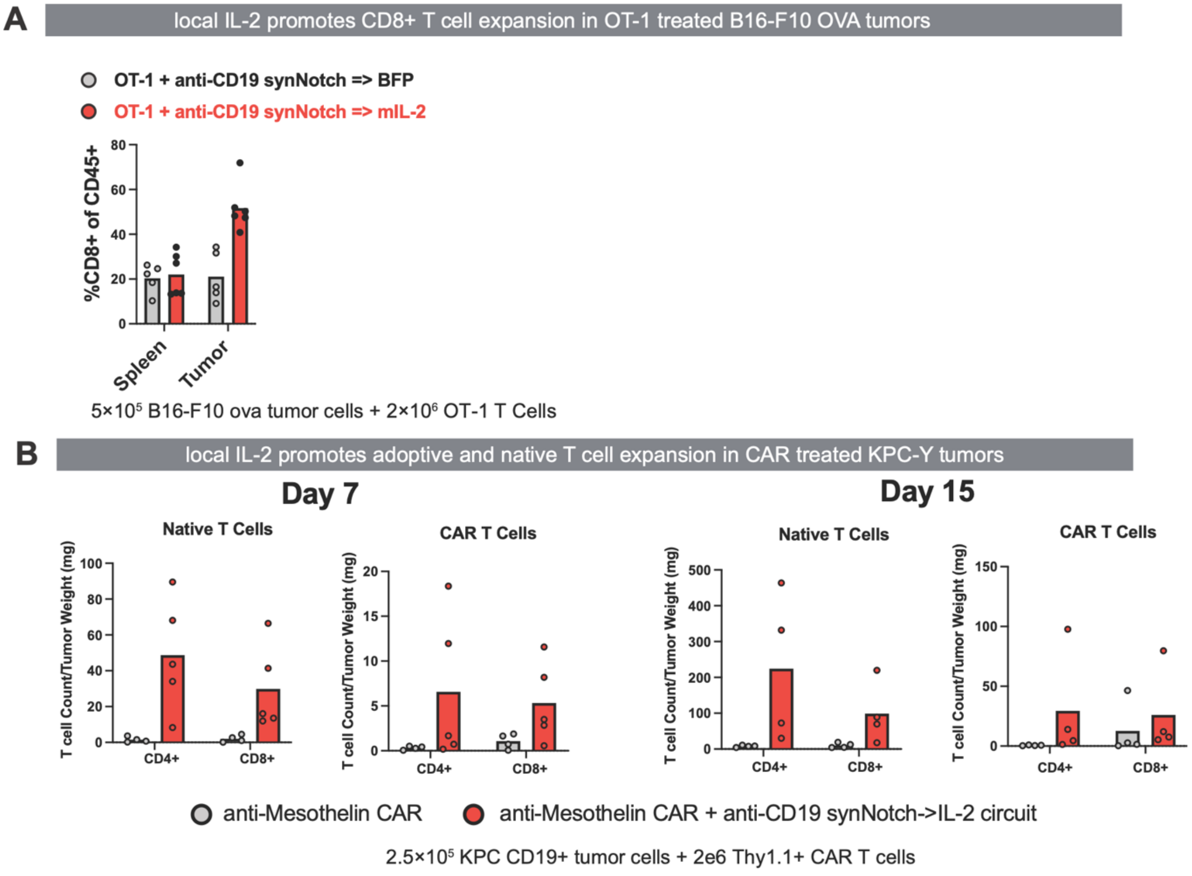

Fig. S12. Enhanced local infiltration of T cells in immunocompotent melanoma and pancreatic tumors

(A) B16-F10 OVA tumors expressing the synNotch target ligand CD19 were treated with 2e6 dose of OT-1 T cells 8 days after tumor implantation. T cells were engineered with anti-huCD19 SynNotch expressing either mIL2 (synthetic cytokine circuit) or irrelevant payload (effLuc or BFP). Tumor size was monitored, and tumors and spleens were collected at indicated endpoint and fraction of $CD45^{+}$ cells expressing CD8 were calculated.

(B) KPC tumors were engrafted sub-cutaneously and treated on tumor day 9 with 2e6 engineered CD3+ mouse T cells. Tumors were collected 7 days and 15 days after T cell treatment and analyzed by flow cytometry for T cell infiltration (CD4 and CD8) from either native (Thy1.2) or adoptive/CAR (Thy1.1) origin. Data is plotted as T cell count per milligram of tumor analyzed.

Supplemental Figure 13 (assoc with Figure 5)

A

mean marker expression for clusters used in phenograph plots in Figure 5

CyTOF PROFILING OF KPC PANCREATIC TUMORS
Unsupervised clustering, Day 9 after treatment, Mean marker expression for each Phenograph cluster
Matching Figure 4C.

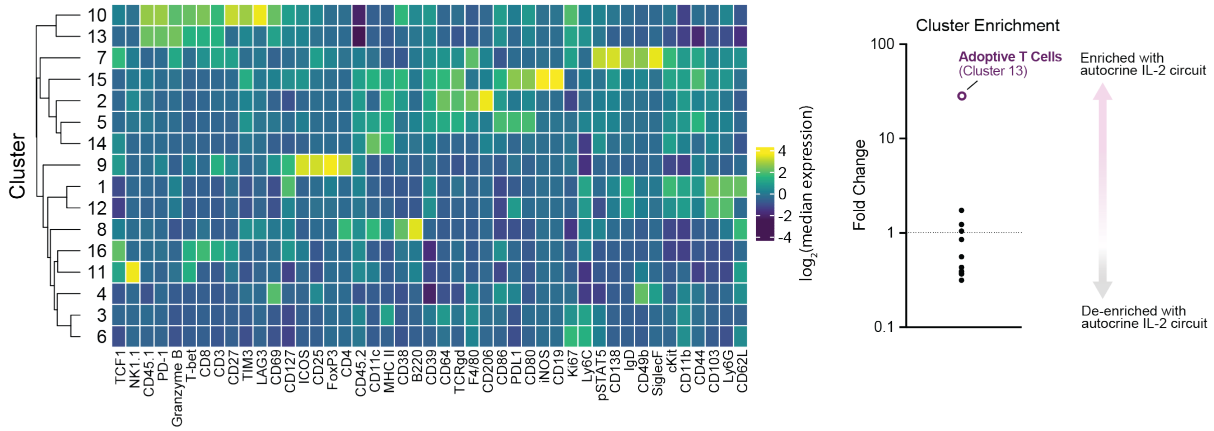

B

Autocrine IL-2 circuit shows enrichment of activated CAR T cells not seen with paracrine circuit.

CyTOF PROFILING OF KPC PANCREATIC TUMORS (AUTOCRINE VS PARACRINE SYNNOTCH-IL-2 CIRCUITS)
Unsupervised clustering, Day 9 after treatment
Paracrine IL-2 circuit vs Autocrine IL-2 circuit (mean marker expression as above)

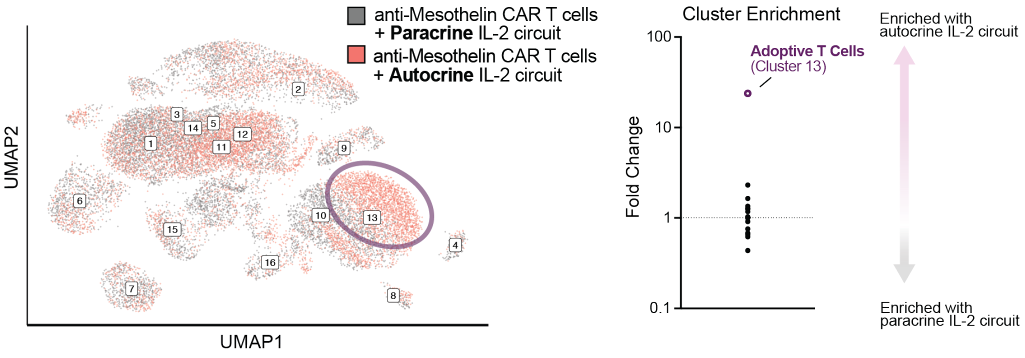

Fig. S13. CyTOF analysis of autocrine and paracrine IL-2 circuits in KPC tumors.
(**A**) As in Figure 4, KPC CD19^{+} tumors were engrafted sub-cutaneously and treated on tumor day 9 with 2e6 engineered CD3^{+} T cells as labelled. Tumors were collected 9 days after T cell treatment and analyzed by CyTOF. Shown is a heatmap with marker express for individual unsupervised clusters by Phenograph of CD45^{+} cells collected from the tumors (**see Figure 5B**). Marker expression is shown as the log 2 of median expression (normalized to the median of each marker). Enrichment was only seen in adoptively transferred CAR T cells when the synthetic IL-2 circuit was engaged (purples circles, $P < 0.0001$ one-way ANOVA).
(**B**) Here a UMAP spatial projection is shown comparing anti-Mesothelin CAR T cells with a paracrine IL-2 circuit (a second cell is engineered with the synNotch => mIL-2 circuit) to the autocrine circuit (the same cell expressess both synNotch and CAR receptors). The autocrine circuit shows enrichment of the adoptive T cells only (see panel A for mean marker expression fo reach phenography cluster).

Supplemental Figure 14 (assoc with Figure 5)

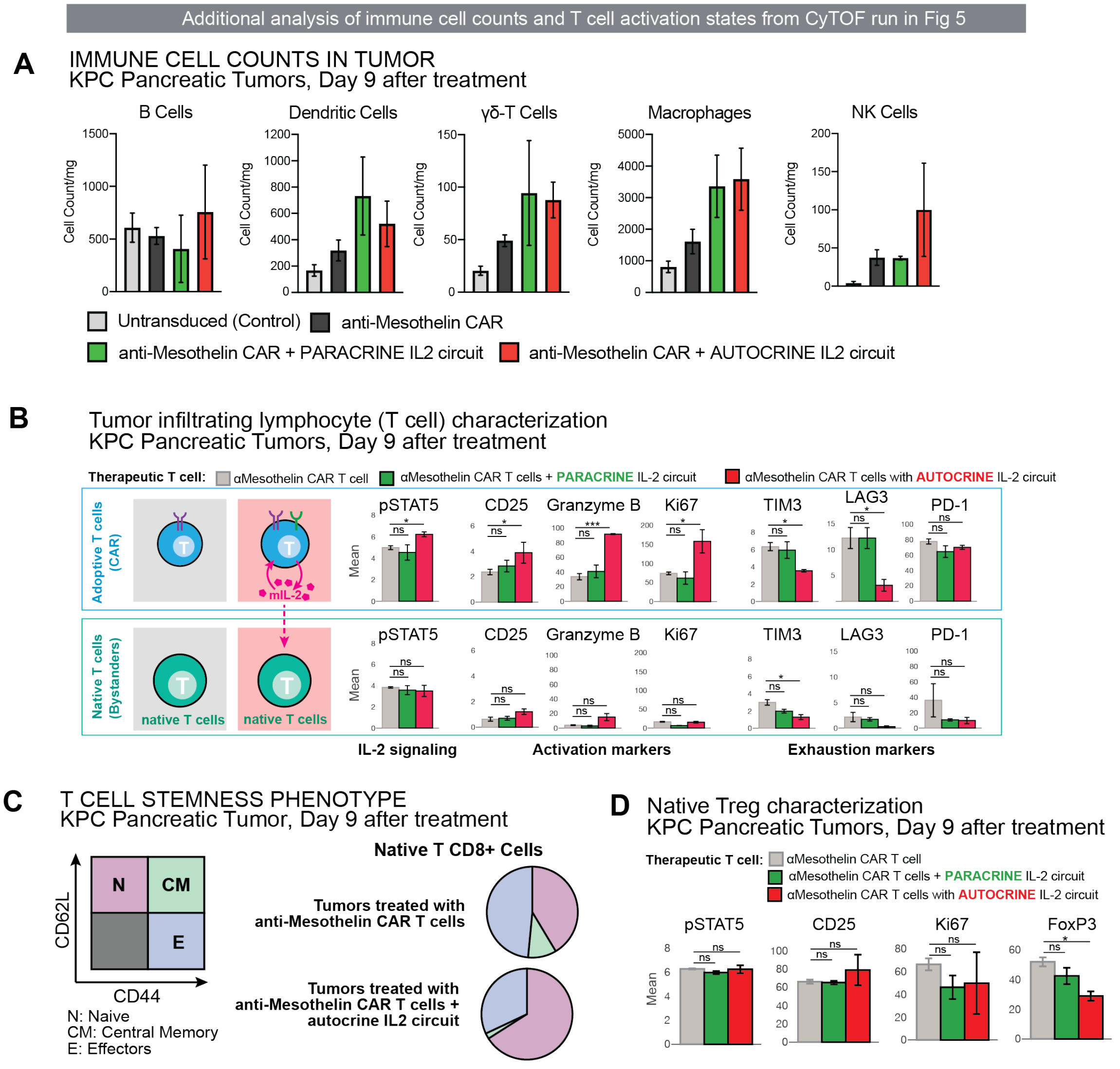

Fig. S14. CyTOF analysis of treated KPC pancreatic tumors

(**A**) As in Figure 4, KPC $CD19^+$ tumors were engrafted sub-cutaneously and treated on tumor day 9 with 2e6 engineered $CD3^+$ T cells as labelled. Tumors were collected 8 days after T cell treatment and analyzed by CyTOF. Shown is cell counts as determined by manual gating (n=3). All counts are normalized by tumor weight, compare to **Figure 5A**.

(**B**) KPC $CD19^+$ tumors were analyzed by CyTOF as in (*A*). IL-2 signaling markers (pSTAT5), activation markers (CD25, Granzyme B, Ki67) and exhaustion markers (CD39, Tim3, Lag3, PD-1) in adoptive/CAR T cells (CD45.1) compared to native/endogenous T cells (CD45.2). Adoptive T cells show increased IL-2 signaling, activaiton, and reduced exhaustion when combined with a synthetic IL-2 circuit in autocrine but not paracrine. Native T cells shows minimal activation or exhaustion in all circuits. Statistical significance was tested using a two-tailed Student's t test [not significant (ns) > 0.05, *P < 0.05, ***P < 0.001] comparing to aMesothelin CAR T cell only.

(**C**) KPC $CD19^+$ tumors were analyzed by CyTOF as in (*A*). Native T cell phenotype was analyzed and and showed that the majority of native T cells are in a naive (non-antigen experienced phenotype) state when a synthetic IL-2 circuit was administered.

(**D**) KPC $CD19^+$ tumors were analyzed by CyTOF as in (*A*). Markers (pSTAT5, CD25, Ki67, FoxP3) in native Tregs are shown. Statistical significance was tested using a two-tailed Student's t test [not significant (ns) > 0.05, *P < 0.05]

Supplemental Figure 15 (assoc with Figure 5)

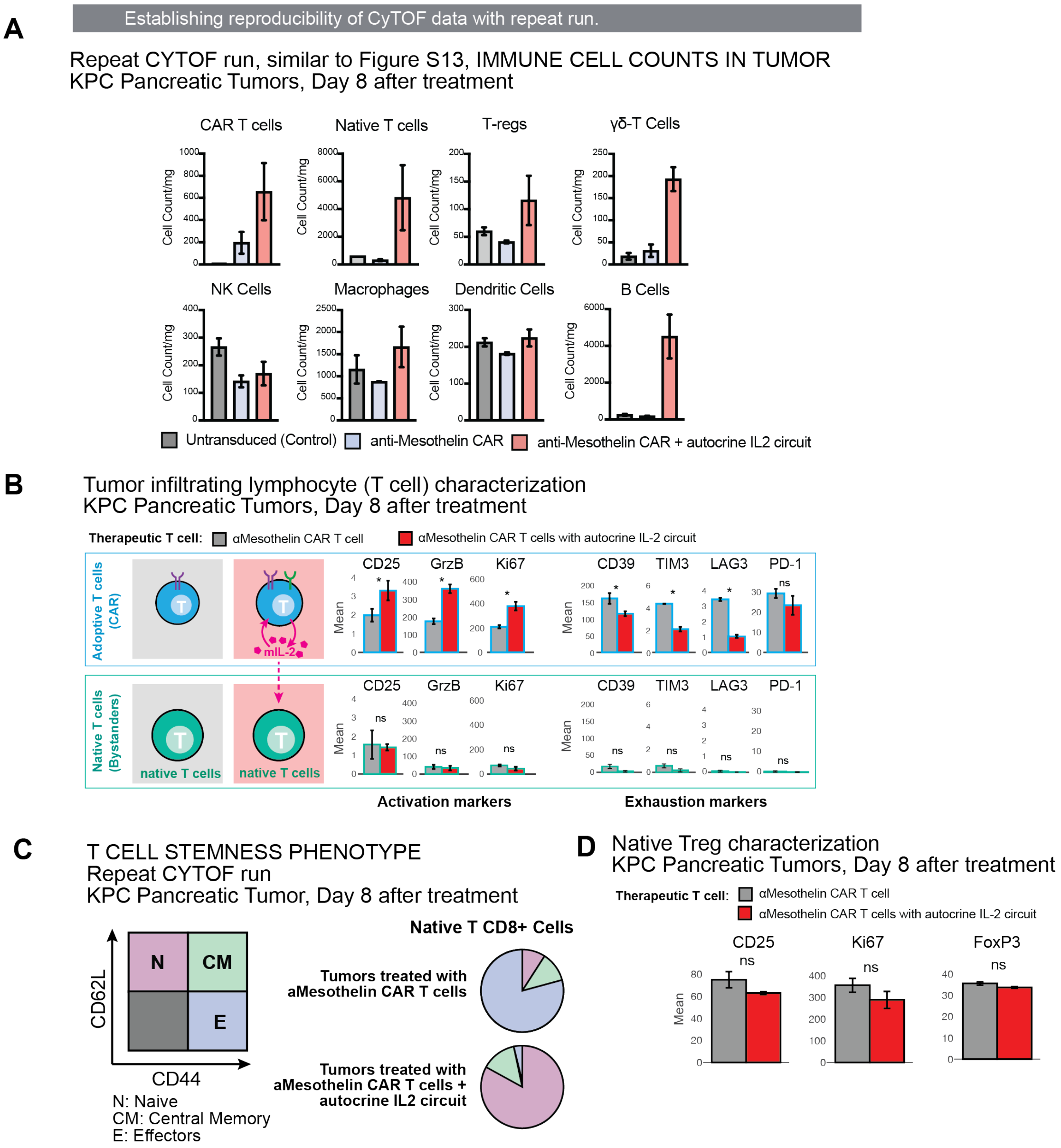

Fig. S15. Repeat CyTOF analysis of treated KPC pancreatic tumors
(**A**) As in **Sup Fig 14**, KPC CD19+ tumors were engrafted sub-cutaneously and treated on tumor day 9 with 2e6 engineered CD3+ T cells as labelled. On this repeat experiment tumors were collected 8 days after T cell treatment and analyzed by CyTOF. Shown are cell counts that were determined by manual gating (n=2). All counts are normalized by tumor weight.
(**B**) KPC CD19+ tumors were analyzed by CyTOF as in (*A*). Exhaustion markers (CD39, Tim3, Lag3, PD-1) showed low expression on endogenous CD8+ T cells.Statistical significance was tested using a two-tailed Student's t test [not significant (ns) > 0.05, *P < 0.05, ***P < 0.001].
(**C**) KPC CD19+ tumors were analyzed by CyTOF as in (*A*). Native T cell phenotype was analyzed and and showed that the majority of native T cells are in a naive (non-antigen experienced phenotype) state when a synthetic IL-2 circuit was administered.
(**D**) KPC CD19^{+} tumors were analyzed by CyTOF as in (*A*). Markers (pSTAT5, CD25, Ki67, FoxP3) in native Tregs are shown. Statistical significance was tested using a two-tailed Student's t test [not significant (ns) > 0.05, *P < 0.05]

Supplemental Figure 16 (assoc with Figure 5)

CyTOF analysis of spleens show no change with synthetic IL-2 circuit

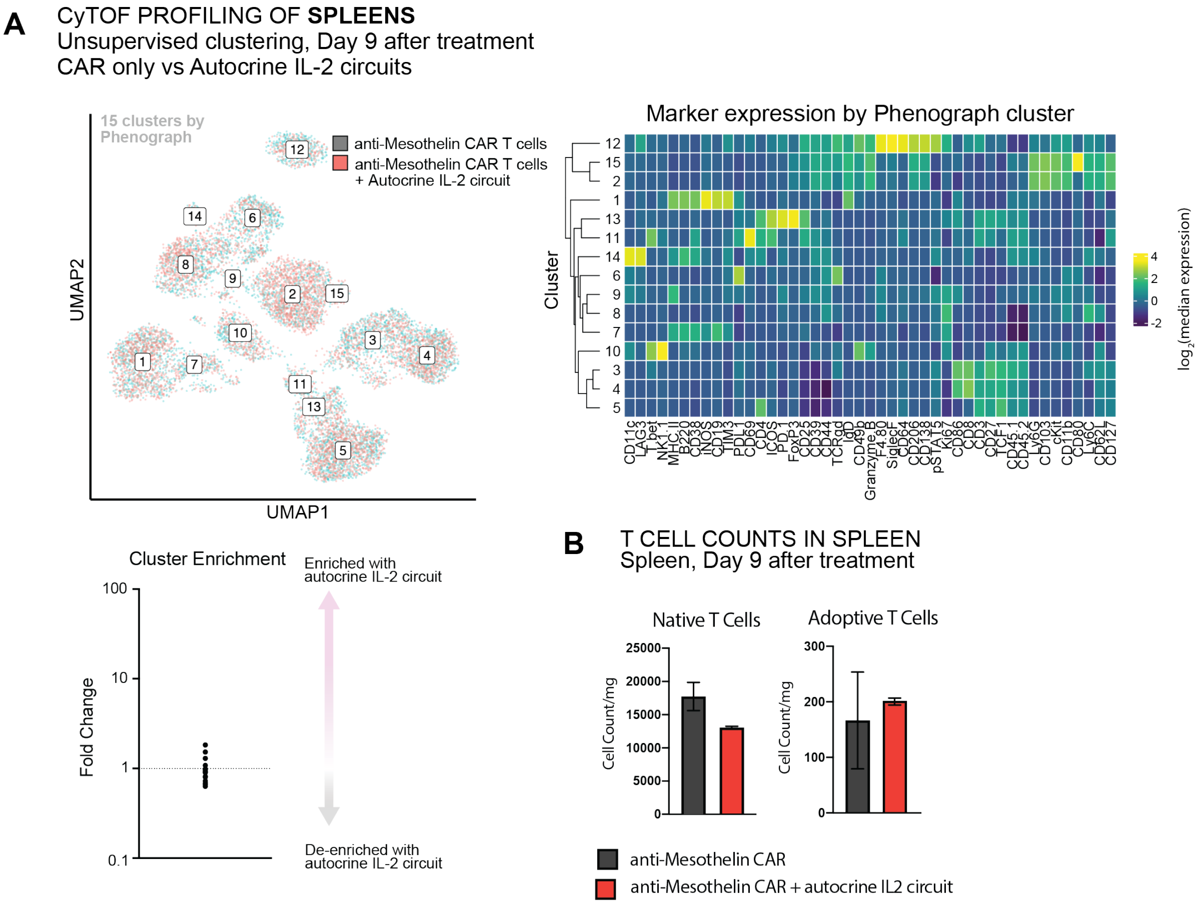

Fig. S16. CyTOF analysis of spleens in mice treated .
(**A**) As in Figure 5, KPC CD19$^+$ tumors were engrafted sub-cutaneously and treated on tumor day 9 with 2e6 engineered CD3$^+$ T cells as labelled. Spleens were collected 9 days after T cell treatment and analyzed by CyTOF. Shown is unsupervised analysis of CyTOF data from the spleens. UMAP shown for spleens in mice treated by anti-Mesothelin CAR +/- IL-2 circuit (autocrine). Labelled numbers indicate clusters by Phenograph. a heatmap with marker express for individual unsupervised clusters by Phenograph of CD45$^+$ cells collected from the spleens is on the right. Marker expression is shown as the log 2 of median expression (normalized to the median of each marker).
(**B**) CyTOF counts of native (CD45.2)+ and adoptive/CAR (CD45.1)+ T cells in the spleens of mice as in (*panel A*) shows no proliferation of T cells outside the tumor micro-environment.

Supplemental Figure 17 (assoc with Figure 6)

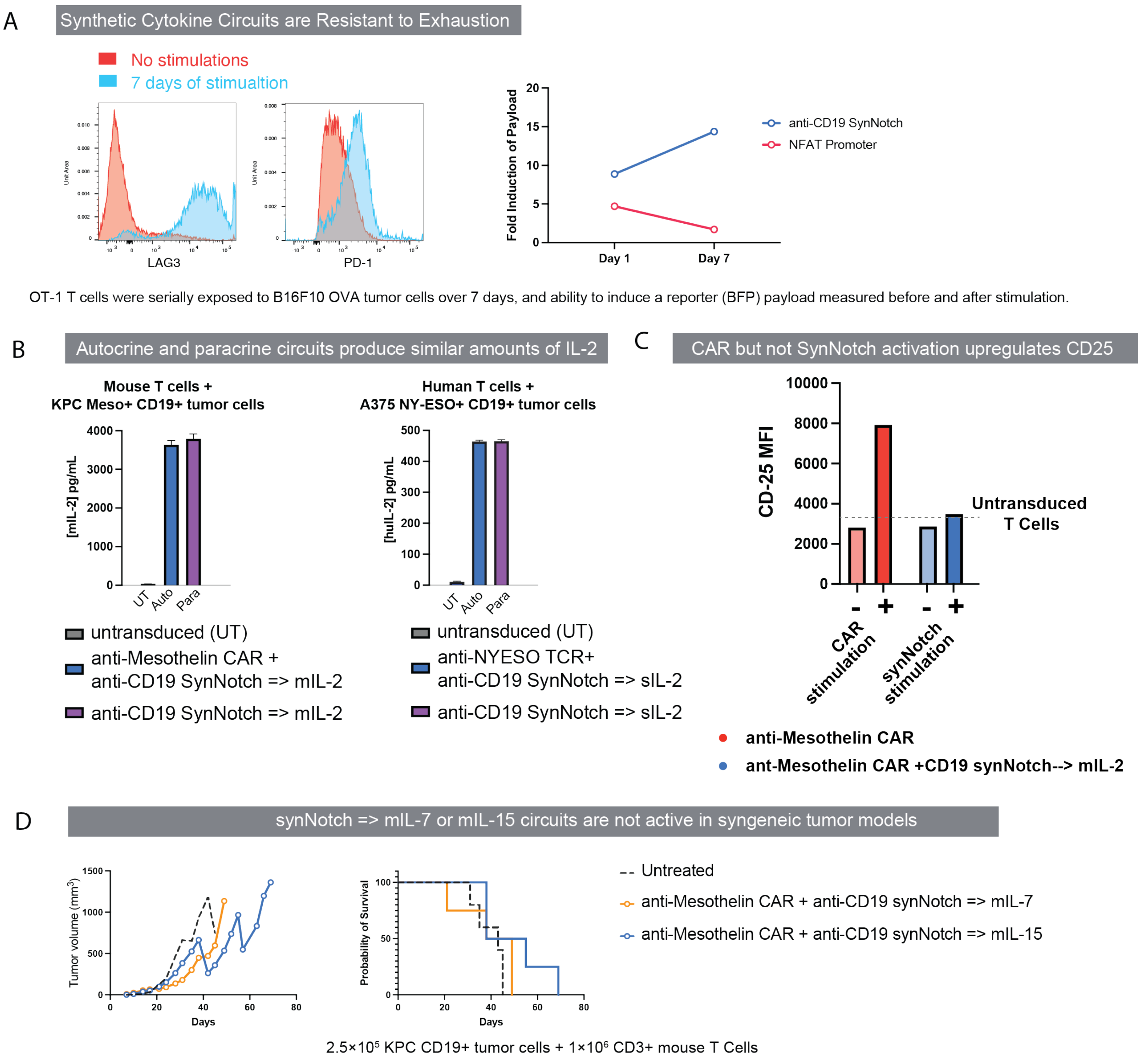

Fig. S17. Estimation of synthetic cytokine circuit strength and induction of CD25

(A) OT-1 T cells with either an anti-CD19 SynNotch => BFP or pNFAT => BFP circuit were co-cultured with B16F10 OVA CD19+ tumor cells for 7 days at a 1:1 ratio. Every 2 days T cells were collected, counted, and re-challenged with fresh B16-F10 OVA CD19+ tumor cells to induce T cell exhaustion. After 7 days exhaustion was measured by cell surface staining for Lag3 and PD1 (as shown) and ability of inducible promoters to drive targeted payload (BFP) measured by flow cytometry.

(B) 50,000 mouse T cells as indicated were co-culutred with 50,000 CD19+ KPC tumor cells and supernatent collected and anlysed by ELISA for expression of mouse IL-2. For human T cells experiments: 50,000 human T cells as indicated were co-cultured with 50,000 CD19+ A375 tumor cells and supernatent collected and analysed by ELISA for expression of human IL-2.

(C) 50,000 mouse T cells as indicated were cultured with or without stimulation for 48 hours and CD25 expression measured by flow cytometry. CAR T cells were stimulated with 50,000 CD19+ KPC tumor cells; myc-tagged SynNotch T cells were stimulated with anti-myc beads. CD25 was only upregulated when CAR was activated.

(D) Mouse T cells were engineered with an anti-CD19 SynNotch => mIL-7 or anti-CD19 SynNotch => mIL-15 circuit as labelled and used to treat sub-cutaneously engrafted KPC CD19+ tumors (as in Figure 2). On left tumor growth curves are shown and on the right mouse survival is plotted.

53. B. M. Allen, K. J. Hiam, C. E. Burnett, A. Venida, R. DeBarge, I. Tenvooren, D. M. Marquez, N. W. Cho, Y. Carmi, M. H. Spitzer, Systemic dysfunction and plasticity of the immune macroenvironment in cancer models. *Nat. Med.* **26**, 1125–1134 (2020).

Table S1.

**Name (all in pHR backbone)**	**Order of Elements (:: indicates same transcript)**
anti-CD19 synNotch	pGK promoter, Kozak sequence, CD8 signal sequence::Myc-tag::anti-CD19-scFv::synNotch GAL4VP64
GAL4UAS hIL-2 IRES mCherry	GAL4UAS, human IL-2, IRES, mCherry
GAL4UAS hIL-7 IRES mCherry	GAL4UAS, human IL-7, IRES, mCherry
GAL4UAS hIL-15 IRES mCherry	GAL4UAS, human IL-15, IRES, mCherry
GAL4UAS super-2 IRES mCherry	GAL4UAS, super-2, IRES, mCherry
GAL4UAS tagBFP P2A super-2 pGK anti-CD19 synNotch	GAL4UAS, Kozak sequence, tagBFP::P2A-Furin::super-2, pGK promoter, Kozak sequence, CD8 signal sequence::Myc-tag::anti-CD19-scFv::synNotch GAL4VP64
GAL4UAS mCherry P2A super-2 pGK anti-GFP synNotch	GAL4UAS, Kozak sequence, mCherry::P2A-Furin::super-2, pGK promoter, Kozak sequence, CD8 signal sequence::Myc-tag::LaG16-LaG2::synNotch GAL4VP64
SFFV affinity-matured anti-NY-ESO TCR	SFFV promoter, tagBFP::P2A-Furin::affinity-matured anti-NY-ESO-1 TCR beta chain::T2A-V5-tag-Furin::affinity-matured anti-NY-ESO-1 TCR alpha chain
SFFV eff-luc P2A mCherry	SFFV promoter, Kozak sequence, enhanced fire-fly luciferase::P2A-Furin::mCherry
SFFV eGFP	SFFV promoter, Kozak sequence, eGFP

SFFV CD19-ligand	SFFV promoter, Kozak sequence, IgKappa signal sequence::CD19 ECD::PDGFRb TMD
SFFV GFP-ligand	SFFV promoter, Kozak sequence, IgKappa signal sequence::eGFP::PDGFRb TMD

Constituent components of pHR lentiviral vectors

Table S2.
Sequences of components referenced in Supplementary Table 1.

**Element name**	**sequence**
Anti-CD19-scFv	DIQMTQTTSSLSASLGDRVTISCRASQDISKYLNWYQQKPDGTV KLLIYHTSRLHSGVPSRFSGSGSGTDYSLTISNLEQEDIATYFCQQ GNTLPYTFGGGTKLEITGGGGSGGGGSGGGGSEVKLQESGPGLV APSQSLSVTCTVSGVSLPDYGVSWIRQPPRKGLEWLGVIWGSETT YYNSALKSRLTIIKDNSKSQVFLKMNSLQTDDTAIYYCAKHYYYG GSYAMDYWGQGTSVTVSS
LaG16-LaG2 (tandem nanobody)	MAQVQLVESGGRLVQAGDSLRLSCAASGRTFSTSAMAWFRQAP GREREFVAAITWTVGNTILGDSVKGRFTISRDRAKNTVDLQMDN LEPEDTAVYYCSARSRGYVLSVLRSVDSYDYWGQGTQVTVSGG GGSGGGGSGGGGSMAQVQLVESGGGLVQAGGSLRLSCAASGRT FSNYAMGWFRQAPGKEREFVAAISWTGVSTYYADSVKGRFTISR DNDKNTVYVQMNSLIPEDTAIYYCAAVRARSFSDTYSRVNEYDY WGQGTQVTV
CD8 signal sequence	MALPVTALLLPLALLLHAARP
synNotch GAL4VP64	ILDYSFTGGAGRDIPPPQIEEACELPECQVDAGNKVCNLQCNNHA CGWDGGDCSLNFNDPWKNCTQSLQCWKYFSDGHCDSQCNSAG CLFDGFDCQLTEGQCNPLYDQYCKDHFSDGHCDQGCNSAECEW DGLDCAEHVPERLAAGTLVLVVLLPPDQLRNNSFHFLRELSHVL HTNVVFKRDAQGQQMIFPYYGHEEELRKHPIKRSTVGWATSSLL PGTSGGRQRRELDPMDIRGSIVYLEIDNRQCVQSSSQCFQSATDV AAFLGALASLGSLNIPYKIEAVKSEPVEPPLPSQLHLMYVAAAAF VLLFFVGCGVLLSRKRRRMKLLSSIEQACDICRLKKLKCSKEKPK CAKCLKNNWECRYSPKTKRSPLTRAHLTEVESRLERLEQLFLLIF PREDLDMILKMDSLQDIKALLTGLFVQDNVNKDAVTDRLASVET DMPLTLRQHRISATSSSEESSNKGQRQLTVSAAAGGSGGSGGSDA LDDFDLDMLGSDALDDFDLDMLGSDALDDFDLDMLGSDALDDF DLDMLGS
GAL4UAS (5x Gal4 response elements with mCMV promoter)	GGAGCACTGTCCTCCGAACGTCGGAGCACTGTCCTCCGAACGT CGGAGCACTGTCCTCCGAACGTCGGAGCACTGTCCTCCGAACG GAGCATGTCCTCCGAACGTCGGAGCACTGTCCTCCGAACGACT AGTTAGGCGTGTACGGTGGGAGGCCTATATAAGCAGAGCTCGT TTAGTGAACCGTCAGATCGCCTGGAGACGCCATCCACGCTGTTT TGACCTCCATAGAAGACACCGGGACCGATCCAGC
SFFV promoter (constitutive)	CCGATAAAATAAAAGATTTTATTTAGTCTCCAGAAAAAGGGG GGAATGAAAGACCCCACCTGTAGGTTTGGCAAGCTAGCTGCA GTAACGCCATTTTGCAAGGCATGGAAAAATACCAAACCAAGA ATAGAGAAGTTCAGATCAAGGGCGGGTACATGAAAATAGCTA ACGTTGGGCCAAACAGGATATCTGCGGTGAGCAGTTTCGGCCC CGGCCCGGGGCCAAGAACAGATGGTCACCGCAGTTTCGGCCCC GGCCCGAGGCCAAGAACAGATGGTCCCCAGATATGGCCCAACC CTCAGCAGTTTCTTAAGACCCATCAGATGTTTCCAGGCTCCCCC AAGGACCTGAAATGACCCTGCGCCTTATTTGAATTAACCAATC AGCCTGCTTCTCGCTTCTGTTCGCGCGCTTCTGCTTCCCGAGCT CTATAAAAGAGCTCACAACCCCTCACTCGGCGCGCCAGTCCTC CGACAGACTGAGTCGCCCGGG

pGK promoter (constitutive)	GGGTAGGGGAGGCGCTTTTCCCAAGGCAGTCTGGAGCATGC GCTTTAGCAGCCCCGCTGGGCACTTGGCGCTACACAAGTGG CCTCTGGCCTCGCACACATTCCACATCCACCGGTAGGCGCC AACCGGCTCCGTTCTTTGGTGGCCCCTTCGCGCCACCTTCTA CTCCTCCCCTAGTCAGGAAGTTCCCCCCCGCCCCGCAGCTCG CGTCGTGCAGGACGTGACAAATGGAAGTAGCACGTCTCACT AGTCTCGTGCAGATGGACAGCACCGCTGAGCAATGGAAGCG GGTAGGCCTTTGGGGCAGCGGCCAATAGCAGCTTTGCTCCTT CGCTTTCTGGGCTCAGAGGCTGGGAAGGGGTGGGTCCGGGG GCGGGCTCAGGGGCGGGCTCAGGGGCGGGGCGGGCGCCCG AAGGTCCTCCGGAGGCCCGGCATTCTGCACGCTTCAAAAGC GCACGTCTGCCGCGCTGTTCTCCTCTTCCTCATCTCCGGGCC TTTCG
Kozak sequence	gccgccacc
Myc-tag	EQKLISEEDL
Human IL-2	MYRMQLLSCIALSLALVTNSAPTSSSTKKTQLQLEHLLLDLQ MILNGINNYKNPKLTRMLTFKFYMPKKATELKHLQCLEEEL KPLEEVLNLAQSKNFHLRPRDLISNINVIVLELKGSETTFMCE YADETATIVEFLNRWITFCQSIISTLT
Human IL-7	
Human IL-15	MRISKPHLRSISIQCYLCLLLNSHFLTEAGIHVFILGCFSAGLP KTEANWVNVISDLKKIEDLIQSMHIDATLYTESDVHPSCKVT AMKCFLLELQVISLESGDASIHDTVENLIILANNSLSSNGNVT ESGCKECEELEEKNIKEFLQSFVHIVQMFINTS
Super-2	MYRMQLLSCIALSLALVTNSAPTSSSTKKTQLQLEHLLLDLQ MILNGINNYKNPKLTRMLTFKFYMPKKATELKHLQCLEEELK PLEEVLNLAQSKNFHFDPRDVVSNINVFVLELKGSETTFMCE YADETATIVEFLNRWITFCQSIISTLT
IRES	CCCCTCTCCCTCCCCCCCCCCTAACGTTACTGGCCGAAGCCG CTTGGAATAAGGCCGGTGTGCGTTTGTCTATATGTTATTTTC CACCATATTGCCGTCTTTTGGCAATGTGAGGGCCCGGAAAC CTGGCCCTGTCTTCTTGACGAGCATTCCTAGGGGTCTTTCCC CTCTCGCCAAAGGAATGCAAGGTCTGTTGAATGTCGTGAAG GAAGCAGTTCCTCTGGAAGCTTCTTGAAGACAAACAACGTC TGTAGCGACCCTTTGCAGGCAGCGGAACCCCCCACCTGGCG ACAGGTGCCTCTGCGGCCAAAAGCCACGTGTATAAGATACA CCTGCAAAGGCGGCACAACCCCAGTGCCACGTTGTGAGTTG GATAGTTGTGGAAAGAGTCAAATGGCTCTCCTCAAGCGTAT TCAACAAGGGGCTGAAGGATGCCCAGAAGGTACCCCATTGT ATGGGATCTGATCTGGGGCCTCGGTGCACATGCTTTACATGT GTTTAGTCGAGGTTAAAAAAACGTCTAGGCCCCCCGAACCA CGGGGACGTGGTTTTCCTTTGAAAAACACGATGATAA
P2A-Furin	RKRRSGSGATNFSLLKQAGDVEENPGP
T2A-V5-tag-Furin	RKRRGKPIPNPLLGLDSTSGSGEGRGSLLTCGDVEENPGP
tagBFP	MSELIKENMHMKLYMEGTVDNHHFKCTSEGEGKPYEGTQTMRI KVVEGGPLPFAFDILATSFLYGSKTFINHTQGIPDFFKQSFPEGFT WERVTTYEDGGVLTATQDTSLQDGCLIYNVKIRGVNFTSNGPV

	MQKKTLGWEAFTETLYPADGGLEGRNDMALKLVGGSHLIANI KTTYRSKKPAKNLKMPGVYYVDYRLERIKEANNETYVEQHEV AVARYCDLPSKLGHKLN
mCherry	MVSKGEEDNMAIIKEFMRFKVHMEGSVNGHEFEIEGEGEGRPYE GTQTAKLKVTKGGPLPFAWDILSPQFMYGSKAYVKHPADIPDYL KLSFPEGFKWERVMNFEDGGVVTVTQDSSLQDGEFIYKVKLRGT NFPSDGPVMQKKTMGWEASSERMYPEDGALKGEIKQRLKLKDG GHYDAEVKTTYKAKKPVQLPGAYNVNIKLDITSHNEDYTIVEQY ERAEGRHSTGGMDELYK
eGFP	MVSKGEELFTGVVPILVELDGDVNGHKFSVSGEGEGDATYGKLT LKFICTTGKLPVPWPTLVTTLTYGVQCFSRYPDHMKQHDFFKSA MPEGYVQERTIFFKDDGNYKTRAEVKFEGDTLVNRIELKGIDFK EDGNILGHKLEYNYNSHNVYIMADKQKNGIKVNFKIRHNIEDGS VQLADHYQQNTPIGDGPVLLPDNHYLSTQSALSKDPNEKRDHM VLLEFVTAAGITLGMDELYK
Enhanced fire-fly luciferase	MEDAKNIKKGPAPFYPLEDGTAGEQLHKAMKRYALVPGTIAFTD AHIEVDITYAEYFEMSVRLAEAMKRYGLNTNHRIVVCSENSLQFF MPVLGALFIGVAVAPANDIYNERELLNSMGISQPTVVFVSKKGLQ KILNVQKKLPIIQKIIIMDSKTDYQGFQSMYTFVTSHLPPGFNEYDF VPESFDRDKTIALIMNSSGSTGLPKGVALPHRTACVRFSHARDPIF GNQIIPDTAILSVVPFHHGFGMFTTLGYLICGFRVVLMYRFEEELF LRSLQDYKIQSALLVPTLFSFFAKSTLIDKYDLSNLHEIASGGAPLS KEVGEAVAKRFHLPGIRQGYGLTETTSAILITPEGDDKPGAVGKV VPFFEAKVVDLDTGKTLGVNQRGELCVRGPMIMSGYVNNPEAT NALIDKDGWLHSGDIAYWDEDEHFFIVDRLKSLIKYKGYQVAPA ELESILLQHPNIFDAGVAGLPDDDAGELPAAVVVLEHGKTMTEK EIVDYVASQVTTAKKLRGGVVFVDEVPKGLTGKLDARKIREILIK AKKGGK
affinity-matured anti-NY-ESO-1 TCR beta chain	MSIGLLCCAALSLLWAGPVNAGVTQTPKFQVLKTGQSMTLQCA QDMNHEYMSWYRQDPGMGLRLIHYSVGAGITDQGEVPNGYNV SRSTTEDFPLRLLSAAPSQTSVYFCASSYVGNTGELFFGEGSRLTV LEDLNKVFPPEVAVFEPSEAEISHTQKATLVCLATGFFPDHVELSW WVNGKEVHSGVSTDPQPLKEQPALNDSRYCLSSRLRVSATFWQN PRNHFRCQVQFYGLSENDEWTQDRAKPVTQIVSAEAWGRADCGF TSVSYQQGVLSATILYEILLGKATLYAVLVSALVLMAMVKRKDF
affinity-matured anti-NY-ESO-1 TCR alpha chain	METLLGLLILWLQLQWVSSKQEVTQIPAALSVPEGENLVLNCSFT DSAIYNLQWFRQDPGKGLTSLLLIQSSQREQTSGRLNASLDKSSG RSTLYIAASQPGDSATYLCAVRPLYGGSYIPTFGRGTSLIVHPPNI QNPDPAVYQLRDSKSSDKSVCLFTDFDSQTNVSQSKDSDVYITD KTVLDMRSMDFKSNSAVAWSNKSDFACANAFNNSIIPEDTFFPS PESSCDVKLVEKSFETDTNLNFQNLSVIGFRILLLKVAGFNLLMT LRLWSS
IgKappa signal sequence	METDTLLLWVLLLWVPGSTGD
CD19 ECD	RPEEPLVVKVEEGDNAVLQCLKGTSDGPTQQLTWSRESPLKP FLKLSLGLPGLGIHMRPLAIWLFIFNVSQQMGGFYLCQPGPPS EKAWQPGWTVNVEGSGELFRWNVSDLGGLGCGLKNRSSEG PSSPSGKLMSPKLYVWAKDRPEIWEGEPPCLPPRDSLNQSLSQ DLTMAPGSTLWLSCGVPPDSVSRGPLSWTHVHPKGPKSLLSL ELKDDRPARDMWVMETGLLLPRATAQDAGKYYCHRGNLTM SFHLEITARPVLWHWLLRTGGWK

PDGFRb TMD	NAVGQDTQEVIVVPHSLPFKVVVISAILALVVLTIISLIILIMLW<br>QKKPR

Table S3.

Name (all in pMIG2 backbone)	Order of Elements (:: indicates same transcript)
anti-Meso CAR	huPGK promoter, Kozak sequence, CD8 signal sequence::V5-tag::anti-mesothelin-nanobody::cd8hinge:cd8transmembrane::41bb::cd3zeta
BFP-p2a-mIL2	huPGK promoter, Kozak sequence, BFP::p2a::mIL2

Constituent components of pMIG2 lentiviral vectors

Table S4.

Sequences of components referenced in Supplementary Table 3.

Element name	sequence
huPGK promoter	ggggttggggttgcgccttttccaaggcagccctgggtttgcgcagggacgcggctgctctg<br>ggcgtggttccgggaaacgcagcggcgccgaccctgggtctcgcacattcttcacgtccgtt<br>cgcagcgtcacccggatcttcgccgctacccttgtgggccccccggcgacgcttcctgctccg<br>cccctaagtcgggaaggttccttgcggttcgcggcgtgccggacgtgacaaacggaagccg<br>cacgtctcactagtaccctcgcagacggacagcgccagggagcaatggcagcgcgccgac<br>cgcgatgggctgtggccaatagcggctgctcagcagggcgcgccgagagcagcggccggg<br>aaggggcggtgcgggaggcggggtgtggggcggtagtgtgggccctgttcctgcccgcgcg<br>gtgttccgcattctgcaagcctccggagcgcacgtcggcagtcggctccctcgttgaccgaat<br>caccgacctctctccccag
Kozak sequence	gccgcc
CD8 signal sequence	MALPVTALLLPLALLLHAARP
V5-tag	GKPIPNPLLGLDST
Anti-Mesothelin-nanobody	QVQLVQSGGGLVHPGGSLRLSCAASGIDLSLYRMRWYRQAPGKERDLV<br>ALITDDGTSYYEDSVKGRFTITRDNPSNKVFLQMNSLKPEDTAVYYCNAE<br>TPLSPVNYWGQGTQVTVS
Mouse CD8 Hinge + Transmembrane	TTTKPVLRTPSPVHPTGTSQPQRPEDCRPRGSVKGTGLDFACDIYIWAPL<br>AGICVALLLSLIITLICYHRSR

Mouse 41bb costimulatory domain	SVLKWIRKKFPHIFKQPFKKTTGAAQEEDACSCRCPQEEEGGGGGYEL
Mouse CD3z domain	RAKFSRSAETAANLQDPNQLYNELNLGRREEYDVLEKKRARDPEMGGK QQRRRNPQEGVYNALQKDKMAEAYSEIGTKGERRRGKGHDGLYQGLST ATKDTYDALHMQTLAPR
BFP	MSELIKENMHMKLYMEGTVDNHHFKCTSEGEGKPYEGTQTMRIKVVE GGPLPFAFDILATSFLYGSKTFINHTQGIPDFFKQSFPEGFTWERVTTYED GGVLTATQDTSLQDGCLIYNVKIRGVNFTSNGPVMQKKTLGWEAFTETL YPADGGLEGRNDMALKLVGGSHLIANIKTTYRSKKPAKNLKMPGVYYVD YRLERIKEANNETYVEQHEVAVARYCDLPSKLGHKLN
p2a	RKRRSGSGATNFSLLKQAGDVEENPGP
mIL2	MYSMQLASCVTLTLVLLVNSAPTSSSTSSSTAEAQQQQQQQQQQQQH LEQLLMDLQELLSRMENYRNLKLPRMLTFKFYLPKQATELKDLQCLEDEL GPLRHVLDLTQSKSFQLEDAENFISNIRVTVVKLKGSDNTFECQFDDESAT VVDFLRRWIAFCQSIISTSPQ

Table S5.

Name (all in pRetroX backbone)	Order of Elements (:: indicates same transcript)
GAL4UAS BFP huPGK anti-CD19 synNotch	5xGAL4UAS-mCMV, Kozak sequence, BFP, mPGK promoter, Kozak sequence, CD8 signal sequence::Myc-tag::CD19scffv::synNotch GAL4VP64
GAL4UAS mIL-2 huPGK anti-CD19 synNotch	5xGAL4UAS-mCMV, Kozak sequence, mIL2, mPGK promoter, Kozak sequence, CD8 signal sequence::Myc-tag::CD19scfv::synNotch::GAL4VP64
NFAT mIL-2 mPGK GFP	4xNFAT/AP1-mCMV, Kozak sequence, mIL2, mPGK promoter, Kozak sequence, GFP

Constituent components of pRetroX lentiviral vectors

Table S6.

Sequences of components referenced in Supplementary Table 5.

Element name	sequence
GAL4UAS-mCMV	ggagcactgtcctccgaacgtcggagcactgtcctccgaacgtcggagcactgtcctccgaacgtc ggagcactgtcctccgaacgGAGCATGTCCTCCGAACGTCGGAGCACTGTCCTCC GAACGactagttaggcgtgtacggtgggaggcctatataagcagagctcgtttagtgaaccgtc agatcgcctggagacgccatccacgctgttttgacctccatagaagacaccgggaccgatccagc

4xNFAT/AP1-mCMV	AAGAGGAAAATTTGTTTCATACAGAAGGCGTTAAGAGGAAAATTTGTTTCATACAGAAGGCGTTCTAGTAAGAGGAAAATTTGTTTCATACAGAAGGCGTTAAGAGGAAAATTTGTTTCATACAGAAGGCGTTaattctAtaggcgtgtacggtgggaggcctatataagcagagctcgtttagtgaaccgtcagatcgcctggagacgccatccacgctgttttgacctccatagaagacaccgggaccgatccagc
Kozak sequence	gccgccacc
BFP	MSELIKENMHMKLYMEGTVDNHHFKCTSEGEGKPYEGTQTMRIKVVEGGPLPFAFDILATSFLYGSKTFINHTQGIPDFFKQSFPEGFTWERVTTYEDGGVLTATQDTSLQDGCLIYNVKIRGVNFTSNGPVMQKKTLGWEAFTETLYPADGGLEGRNDMALKLVGGSHLIANIKTTYRSKKPAKNLKMPGVYYVDYRLERIKEANNETYVEQHEVAVARYCDLPSKLGHKLN
mIL2	MYSMQLASCVTLTLVLLVNSAPTSSSTSSSTAEAQQQQQQQQQQQQHLEQLLMDLQELLSRMENYRNLKLPRMLTFKFYLPKQATELKDLQCLEDELGPLRHVLDLTQSKSFQLEDAENFISNIRVTVVKLKGSDNTFECQFDDESATVVDFLRRWIAFCQSIISTSPQ
mPGK promoter	GGGTAGGGGAGGCGCTTTTCCCAAGGCAGTCTGGAGCATGCGCTTTAGCAGCCCCGCTGGGCACTTGGCGCTACACAAGTGGCCTCTGGCCTCGCACACATTCCACATCCACCGGTAGGCGCCAACCGGCTCCGTTCTTTGGTGGCCCCTTCGCGCCACCTTCTACTCCTCCCCTAGTCAGGAAGTTCCCCCCCGCCCCGCAGCTCGCGTCGTGCAGGACGTGACAAATGGAAGTAGCACGTCTCACTAGTCTCGTGCAGATGGACAGCACCGCTGAGCAATGGAAGCGGGTAGGCCTTTGGGGCAGCGGCCAATAGCAGCTTTGCTCCTTCGCTTTCTGGGCTCAGAGGCTGGGAAGGGGTGGGTCCGGGGGCGGGCTCAGGGGCGGGCTCAGGGGCGGGGCGGGCGCCCGAAGGTCCTCCGGAGGCCCGGCATTCTGCACGCTTCAAAAGCGCACGTCTGCCGCGCTGTTCTCCTCTTCCTCATCTCCGGGCCTTTCG
CD8 signal sequence	MALPVTALLLPLALLLHAARP
Myc tag	EQKLISEEDL
Anti-CD19scfv	DIQMTQTTSSLSASLGDRVTISCRASQDISKYLNWYQQKPDGTVKLLIYHTSRLHSGVPSRFSGSGSGTDYSLTISNLEQEDIATYFCQQGNTLPYTFGGGTKLEITGGGGSGGGGSGGGGSEVKLQESGPGLVAPSQSLSVTCTVSGVSLPDYGVSWIRQPPRKGLEWLGVIWGSETTYYNSALKSRLTIIKDNSKSQVFLKMNSLQTDDTAIYYCAKHYYYGGSYAMDYWGQGTSVTVSS
synNotch	ILDYSFTGGAGRDIPPPQIEEACELPECQVDAGNKVCNLQCNNHACGWDGGDCSLNFNDPWKNCTQSLQCWKYFSDGHCDSQCNSAGCLFDGFDCQLTEGQCNPLYDQYCKDHFSDGHCDQGCNSAECEWDGLDCAEHVPERLAAGTLVLVVLLPPDQLRNNSFHFLRELSHVLHTNVVFKRDAQGQQMIFPYYGHEEELRKHPIKRSTVGWATSSLLPGTSGGRQRRELDPMDIRGSIVYLEIDNRQCVQSSSQCFQSATDVAAFLGALASLGSLNIPYKIEAVKSEPVEPPLPSQLHLMYVAAAAFVLLFFVGCGVLLSRKRRR
GAL4VP64	MKLLSSIEQACDICRLKKLKCSKEKPKCAKCLKNNWECRYSPKTKRSPLTRAHLTEVESRLERLEQLFLLIFPREDLDMILKMDSLQDIKALLTGLFVQDNVNKDAVTDRLASVETDMPLTLRQHRISATSSSEESSNKGQRQLTVSAAAGGSGGSGGSDALDDFDLDMLGSDALDDFDLDMLGSDALDDFDLDMLGSDALDDFDLDMLGS
GFP	MVSKGEELFTGVVPILVELDGDVNGHKFSVSGEGEGDATYGKLTLKFICTTGKLPVPWPTLVTTLTYGVQCFSRYPDHMKQHDFFKSAMPEGYVQERTIFFKDDGNYKTRAEVKFEGDTLVNRIELKGIDFKEDGNILGHKLEYNYNSHNVYIMADKQKNGIKANFKIRHNIEDGSVQLADHYQQNTPIGDGPVLLPDNHYLSTQSALSKDPNEKRDHMVLLEFVTAAGITLGMDELYK

WPRE	taatcaacctctggattacaaaatttgtgaaagattgactggtattcttaactatgttgctccttttac<br>gctatgtggatacgctgctttaatgcctttgtatcatgctattgcttcccgtatggctttcattttctcct<br>ccttgtataaatcctggttgctgtctctttatgaggagttgtggcccgttgtcaggcaacgtggcgtg<br>gtgtgcactgtgtttgctgacgcaacccccactggttggggcattgccaccacctgtcagctcctttc<br>cgggactttcgctttccccctccctattgccacggcggaactcatcgccgcctgccttgcccgctgct<br>ggacaggggctcggctgttgggcactgacaattccgtggtgttgtcggggaaatcatcgtcctttcc<br>ttggctgctcgcctgtgttgccacctggattctgcgcgggacgtccttctgctacgtcccttcggccct<br>caatccagcggaccttccttcccgcggcctgctgccggctctgcggcctcttccgcgtcttcgccttc<br>gccctcagacgagtcggatctccctttgggccgcctccccgcat

Table S7.
Antibodies for mass cytometry analysis of KPC tumors.

Antigen	Clone	Supplier	Elemental
Ter119	TER119	Biolegend	Y89
CD3	17A2	Biolegend	Cd111
CD64	X54-5/7.1	Biolegend	Cd112
CD45.2	104	Biolegend	In113
CD8	53-6.7	Biolegend	Cd114
CD45.1	A20	Biolegend	In115
CD4	RM4-5	Biolegend	Cd116
Ly6G	1A8	Biolegend	La139
KLRG1	2F1	BD	Ce140
Granzyme B	QA16A02	Biolegend	Pr141
CD49b	HMa2	Biolegend	Nd142
CD11c	N418	Biolegend	Nd143
CD206	C068C2	Biolegend	Nd144
CD27	LG.3A10	Biolegend	Nd145
CD138	281-2	Biolegend	Nd146
PD-1	29F.1A12	Biolegend	Sm147
ICOS	C398.4A	Biolegend	Nd148
SiglecF	E50-2440	BD	Sm149
Ly6C	HK1.4	Biolegend	Eu151
Ki67	SolA15	BD	Sm152
CD11b	M1/70	Biolegend	Eu153
cKit	2B8	Biolegend	Sm154
CD103	2E7	Biolegend	Gd155
B220	RA3-6B2	Biolegend	Gd158
NK1.1	PK136	Biolegend	Gd160
T-bet	O4-46	BD	Dy161
TCRgd	GL3	Biolegend	Dy162

CD62L	95218	R&D	Dy163
TCF1	C63D9	Cell Signal	Dy164
CD69	Polyclonal	R&D	Ho165
CD127	A7R34	Biolegend	Er166
Foxp3	NRRF-30	eBioscience	Er167
CD25	PC61	Biolegend	Er168
F4/80	BM8	Biolegend	Tm169
LAG3	C9B7W	Biolegend	Er170
CD38	90	Biolegend	Yb171
TIM3	RMT3-23	Biolegend	Yb172
CD19	6D5	Biolegend	Yb173
iNOS	CXNFT	eBioscience	Yb174
CD44	IM7	Biolegend	Lu175
CD39	24DMS1	eBioscience	Yb176
MHC II	M5/114.15.2	Biolegend	Bi209
IgD	11-26c.2a	Biolegend	Ce140
CD80	16-10A1	Biolegend	Gd156
CD86	GL-1	Biolegend	Gd157
pSTAT5	47	Fluidigm	Nd150

References and Notes

1. S. J. Schuster, M. R. Bishop, C. S. Tam, E. K. Waller, P. Borchmann, J. P. McGuirk, U. Jäger, S. Jaglowski, C. Andreadis, J. R. Westin, I. Fleury, V. Bachanova, S. R. Foley, P. J. Ho, S. Mielke, J. M. Magenau, H. Holte, S. Pantano, L. B. Pacaud, R. Awasthi, J. Chu, Ö. Anak, G. Salles, R. T. Maziarz; JULIET Investigators, Tisagenlecleucel in Adult Relapsed or Refractory Diffuse Large B-Cell Lymphoma. *N. Engl. J. Med.* **380**, 45–56 (2019). doi:10.1056/NEJMoa1804980 Medline

2. N. C. Munshi, L. D. Anderson Jr., N. Shah, D. Madduri, J. Berdeja, S. Lonial, N. Raje, Y. Lin, D. Siegel, A. Oriol, P. Moreau, I. Yakoub-Agha, M. Delforge, M. Cavo, H. Einsele, H. Goldschmidt, K. Weisel, A. Rambaldi, D. Reece, F. Petrocca, M. Massaro, J. N. Connarn, S. Kaiser, P. Patel, L. Huang, T. B. Campbell, K. Hege, J. San-Miguel, Idecabtagene Vicleucel in Relapsed and Refractory Multiple Myeloma. *N. Engl. J. Med.* **384**, 705–716 (2021). doi:10.1056/NEJMoa2024850 Medline

3. A. R. Haas, J. L. Tanyi, M. H. O'Hara, W. L. Gladney, S. F. Lacey, D. A. Torigian, M. C. Soulen, L. Tian, M. McGarvey, A. M. Nelson, C. S. Farabaugh, E. Moon, B. L. Levine, J. J. Melenhorst, G. Plesa, C. H. June, S. M. Albelda, G. L. Beatty, Phase I Study of Lentiviral-Transduced Chimeric Antigen Receptor-Modified T Cells Recognizing Mesothelin in Advanced Solid Cancers. *Mol. Ther.* **27**, 1919–1929 (2019). doi:10.1016/j.ymthe.2019.07.015 Medline

4. S. Mariathasan, S. J. Turley, D. Nickles, A. Castiglioni, K. Yuen, Y. Wang, E. E. Kadel III, H. Koeppen, J. L. Astarita, R. Cubas, S. Jhunjhunwala, R. Banchereau, Y. Yang, Y. Guan, C. Chalouni, J. Ziai, Y. Şenbabaoğlu, S. Santoro, D. Sheinson, J. Hung, J. M. Giltnane, A. A. Pierce, K. Mesh, S. Lianoglou, J. Riegler, R. A. D. Carano, P. Eriksson, M. Höglund, L. Somarriba, D. L. Halligan, M. S. van der Heijden, Y. Loriot, J. E. Rosenberg, L. Fong, I. Mellman, D. S. Chen, M. Green, C. Derleth, G. D. Fine, P. S. Hegde, R. Bourgon, T. Powles, TGFβ attenuates tumour response to PD-L1 blockade by contributing to exclusion of T cells. *Nature* **554**, 544–548 (2018). doi:10.1038/nature25501 Medline

5. A. Xia, Y. Zhang, J. Xu, T. Yin, X.-J. Lu, T Cell Dysfunction in Cancer Immunity and Immunotherapy. *Front. Immunol.* **10**, 1719 (2019). doi:10.3389/fimmu.2019.01719 Medline

6. A. B. Frey, N. Monu, Signaling defects in anti-tumor T cells. *Immunol. Rev.* **222**, 192–205 (2008). doi:10.1111/j.1600-065X.2008.00606.x Medline

7. M. Y. Balkhi, Q. Ma, S. Ahmad, R. P. Junghans, T cell exhaustion and Interleukin 2 downregulation. *Cytokine* **71**, 339–347 (2015). doi:10.1016/j.cyto.2014.11.024 Medline

8. L. Tang, Y. Zheng, M. B. Melo, L. Mabardi, A. P. Castaño, Y.-Q. Xie, N. Li, S. B. Kudchodkar, H. C. Wong, E. K. Jeng, M. V. Maus, D. J. Irvine, Enhancing T cell therapy through TCR-signaling-responsive nanoparticle drug delivery. *Nat. Biotechnol.* **36**, 707–716 (2018). doi:10.1038/nbt.4181 Medline

9. L. Zhang, R. A. Morgan, J. D. Beane, Z. Zheng, M. E. Dudley, S. H. Kassim, A. V. Nahvi, L. T. Ngo, R. M. Sherry, G. Q. Phan, M. S. Hughes, U. S. Kammula, S. A. Feldman, M. A. Toomey, S. P. Kerkar, N. P. Restifo, J. C. Yang, S. A. Rosenberg, Tumor-infiltrating lymphocytes genetically engineered with an inducible gene encoding interleukin-12 for

the immunotherapy of metastatic melanoma. *Clin. Cancer Res.* **21**, 2278–2288 (2015). doi:10.1158/1078-0432.CCR-14-2085 Medline

10. M. B. Atkins, M. T. Lotze, J. P. Dutcher, R. I. Fisher, G. Weiss, K. Margolin, J. Abrams, M. Sznol, D. Parkinson, M. Hawkins, C. Paradise, L. Kunkel, S. A. Rosenberg, High-dose recombinant interleukin 2 therapy for patients with metastatic melanoma: Analysis of 270 patients treated between 1985 and 1993. *J. Clin. Oncol.* **17**, 2105–2116 (1999). doi:10.1200/JCO.1999.17.7.2105 Medline

11. Q. Zhang, M. E. Hresko, L. K. Picton, L. Su, M. J. Hollander, S. Nunez-Cruz, Z. Zhang, C.-A. Assenmacher, J. T. Sockolosky, K. C. Garcia, M. C. Milone, A human orthogonal IL-2 and IL-2Rβ system enhances CAR T cell expansion and antitumor activity in a murine model of leukemia. *Sci. Transl. Med.* **13**, eabg6986 (2021). doi:10.1126/scitranslmed.abg6986 Medline

12. L. Morsut, K. T. Roybal, X. Xiong, R. M. Gordley, S. M. Coyle, M. Thomson, W. A. Lim, Engineering Customized Cell Sensing and Response Behaviors Using Synthetic Notch Receptors. *Cell* **164**, 780–791 (2016). doi:10.1016/j.cell.2016.01.012 Medline

13. K. T. Roybal, L. J. Rupp, L. Morsut, W. J. Walker, K. A. McNally, J. S. Park, W. A. Lim, Precision Tumor Recognition by T Cells With Combinatorial Antigen-Sensing Circuits. *Cell* **164**, 770–779 (2016). doi:10.1016/j.cell.2016.01.011 Medline

14. K. A. Smith, Interleukin-2: Inception, impact, and implications. *Science* **240**, 1169–1176 (1988). doi:10.1126/science.3131876 Medline

15. Z. Sun, Z. Ren, K. Yang, Z. Liu, S. Cao, S. Deng, L. Xu, Y. Liang, J. Guo, Y. Bian, H. Xu, J. Shi, F. Wang, Y.-X. Fu, H. Peng, A next-generation tumor-targeting IL-2 preferentially promotes tumor-infiltrating $CD8^{+}$ T-cell response and effective tumor control. *Nat. Commun.* **10**, 3874 (2019). doi:10.1038/s41467-019-11782-w Medline

16. G. R. Weiss, W. W. Grosh, K. A. Chianese-Bullock, Y. Zhao, H. Liu, C. L. Slingluff Jr., F. M. Marincola, E. Wang, Molecular insights on the peripheral and intratumoral effects of systemic high-dose rIL-2 (aldesleukin) administration for the treatment of metastatic melanoma. *Clin. Cancer Res.* **17**, 7440–7450 (2011). doi:10.1158/1078-0432.CCR-11-1650 Medline

17. M. C. Panelli, E. Wang, G. Phan, M. Puhlmann, L. Miller, G. A. Ohnmacht, H. G. Klein, F. M. Marincola, Gene-expression profiling of the response of peripheral blood mononuclear cells and melanoma metastases to systemic IL-2 administration. *Genome Biol.* **3**, RESEARCH0035 (2002). doi:10.1186/gb-2002-3-7-research0035 Medline

18. T. Saito, H. Nishikawa, H. Wada, Y. Nagano, D. Sugiyama, K. Atarashi, Y. Maeda, M. Hamaguchi, N. Ohkura, E. Sato, H. Nagase, J. Nishimura, H. Yamamoto, S. Takiguchi, T. Tanoue, W. Suda, H. Morita, M. Hattori, K. Honda, M. Mori, Y. Doki, S. Sakaguchi, Two FOXP3(+)CD4(+) T cell subpopulations distinctly control the prognosis of colorectal cancers. *Nat. Med.* **22**, 679–684 (2016). doi:10.1038/nm.4086 Medline

19. M. Ahmadzadeh, S. A. Rosenberg, IL-2 administration increases CD4+ CD25(hi) Foxp3+ regulatory T cells in cancer patients. *Blood* **107**, 2409–2414 (2006). doi:10.1182/blood-2005-06-2399 Medline

20. K. Staveley-O'Carroll, E. Sotomayor, J. Montgomery, I. Borrello, L. Hwang, S. Fein, D. Pardoll, H. Levitsky, Induction of antigen-specific T cell anergy: An early event in the

course of tumor progression. *Proc. Natl. Acad. Sci. U.S.A.* **95**, 1178–1183 (1998). doi:10.1073/pnas.95.3.1178 Medline

21. A. M. Levin, D. L. Bates, A. M. Ring, C. Krieg, J. T. Lin, L. Su, I. Moraga, M. E. Raeber, G. R. Bowman, P. Novick, V. S. Pande, C. G. Fathman, O. Boyman, K. C. Garcia, Exploiting a natural conformational switch to engineer an interleukin-2 'superkine'. *Nature* **484**, 529–533 (2012). doi:10.1038/nature10975 Medline

22. D. L. Wallace, M. Bérard, M. V. D. Soares, J. Oldham, J. E. Cook, A. N. Akbar, D. F. Tough, P. C. L. Beverley, Prolonged exposure of naïve CD8+ T cells to interleukin-7 or interleukin-15 stimulates proliferation without differentiation or loss of telomere length. *Immunology* **119**, 243–253 (2006). doi:10.1111/j.1365-2567.2006.02429.x Medline

23. L. V. Hurton, H. Singh, A. M. Najjar, K. C. Switzer, T. Mi, S. Maiti, S. Olivares, B. Rabinovich, H. Huls, M.-A. Forget, V. Datar, P. Kebriaei, D. A. Lee, R. E. Champlin, L. J. N. Cooper, Tethered IL-15 augments antitumor activity and promotes a stem-cell memory subset in tumor-specific T cells. *Proc. Natl. Acad. Sci. U.S.A.* **113**, E7788–E7797 (2016). doi:10.1073/pnas.1610544113 Medline

24. P. F. Robbins, Y. F. Li, M. El-Gamil, Y. Zhao, J. A. Wargo, Z. Zheng, H. Xu, R. A. Morgan, S. A. Feldman, L. A. Johnson, A. D. Bennett, S. M. Dunn, T. M. Mahon, B. K. Jakobsen, S. A. Rosenberg, Single and dual amino acid substitutions in TCR CDRs can enhance antigen-specific T cell functions. *J. Immunol.* **180**, 6116–6131 (2008). doi:10.4049/jimmunol.180.9.6116 Medline

25. P. F. Robbins, S. H. Kassim, T. L. N. Tran, J. S. Crystal, R. A. Morgan, S. A. Feldman, J. C. Yang, M. E. Dudley, J. R. Wunderlich, R. M. Sherry, U. S. Kammula, M. S. Hughes, N. P. Restifo, M. Raffeld, C.-C. R. Lee, Y. F. Li, M. El-Gamil, S. A. Rosenberg, A pilot trial using lymphocytes genetically engineered with an NY-ESO-1-reactive T-cell receptor: Long-term follow-up and correlates with response. *Clin. Cancer Res.* **21**, 1019–1027 (2015). doi:10.1158/1078-0432.CCR-14-2708 Medline

26. J. Z. Williams, G. M. Allen, D. Shah, I. S. Sterin, K. H. Kim, V. P. Garcia, G. E. Shavey, W. Yu, C. Puig-Saus, J. Tsoi, A. Ribas, K. T. Roybal, W. A. Lim, Precise T cell recognition programs designed by transcriptionally linking multiple receptors. *Science* **370**, 1099–1104 (2020). doi:10.1126/science.abc6270 Medline

27. C. C. Kloss, M. Condomines, M. Cartellieri, M. Bachmann, M. Sadelain, Combinatorial antigen recognition with balanced signaling promotes selective tumor eradication by engineered T cells. *Nat. Biotechnol.* **31**, 71–75 (2013). doi:10.1038/nbt.2459 Medline

28. G. P. Mognol, R. Spreafico, V. Wong, J. P. Scott-Browne, S. Togher, A. Hoffmann, P. G. Hogan, A. Rao, S. Trifari, Exhaustion-associated regulatory regions in $CD8^+$ tumor-infiltrating T cells. *Proc. Natl. Acad. Sci. U.S.A.* **114**, E2776–E2785 (2017). doi:10.1073/pnas.1620498114 Medline

29. T. Chinen, A. K. Kannan, A. G. Levine, X. Fan, U. Klein, Y. Zheng, G. Gasteiger, Y. Feng, J. D. Fontenot, A. Y. Rudensky, An essential role for the IL-2 receptor in T_{reg} cell function. *Nat. Immunol.* **17**, 1322–1333 (2016). doi:10.1038/ni.3540 Medline

30. G. Oliveira, K. Stromhaug, S. Klaeger, T. Kula, D. T. Frederick, P. M. Le, J. Forman, T. Huang, S. Li, W. Zhang, Q. Xu, N. Cieri, K. R. Clauser, S. A. Shukla, D. Neuberg, S. Justesen, G. MacBeath, S. A. Carr, E. F. Fritsch, N. Hacohen, M. Sade-Feldman, K. J.

Livak, G. M. Boland, P. A. Ott, D. B. Keskin, C. J. Wu, Phenotype, specificity and avidity of antitumour $CD8^+$ T cells in melanoma. *Nature* **596**, 119–125 (2021). doi:10.1038/s41586-021-03704-y Medline

31. J. Li, K. T. Byrne, F. Yan, T. Yamazoe, Z. Chen, T. Baslan, L. P. Richman, J. H. Lin, Y. H. Sun, A. J. Rech, D. Balli, C. A. Hay, Y. Sela, A. J. Merrell, S. M. Liudahl, N. Gordon, R. J. Norgard, S. Yuan, S. Yu, T. Chao, S. Ye, T. S. K. Eisinger-Mathason, R. B. Faryabi, J. W. Tobias, S. W. Lowe, L. M. Coussens, E. J. Wherry, R. H. Vonderheide, B. Z. Stanger, Tumor Cell-Intrinsic Factors Underlie Heterogeneity of Immune Cell Infiltration and Response to Immunotherapy. *Immunity* **49**, 178–193.e7 (2018). doi:10.1016/j.immuni.2018.06.006 Medline

32. S. R. Hingorani, L. Wang, A. S. Multani, C. Combs, T. B. Deramaudt, R. H. Hruban, A. K. Rustgi, S. Chang, D. A. Tuveson, Trp53R172H and KrasG12D cooperate to promote chromosomal instability and widely metastatic pancreatic ductal adenocarcinoma in mice. *Cancer Cell* **7**, 469–483 (2005). doi:10.1016/j.ccr.2005.04.023 Medline

33. T. N. D. Pham, M. A. Shields, C. Spaulding, D. R. Principe, B. Li, P. W. Underwood, J. G. Trevino, D. J. Bentrem, H. G. Munshi, Preclinical Models of Pancreatic Ductal Adenocarcinoma and Their Utility in Immunotherapy Studies. *Cancers* **13**, 440 (2021). doi:10.3390/cancers13030440 Medline

34. I. M. Stromnes, T. M. Schmitt, A. Hulbert, J. S. Brockenbrough, H. Nguyen, C. Cuevas, A. M. Dotson, X. Tan, J. L. Hotes, P. D. Greenberg, S. R. Hingorani, T Cells Engineered against a Native Antigen Can Surmount Immunologic and Physical Barriers to Treat Pancreatic Ductal Adenocarcinoma. *Cancer Cell* **28**, 638–652 (2015). doi:10.1016/j.ccell.2015.09.022 Medline

35. M. K. Gately, T. D. Anderson, T. J. Hayes, Role of asialo-GM1-positive lymphoid cells in mediating the toxic effects of recombinant IL-2 in mice. *J. Immunol.* **141**, 189–200 (1988). Medline

36. E. Hooijberg, A. Q. Bakker, J. J. Ruizendaal, H. Spits, NFAT-controlled expression of GFP permits visualization and isolation of antigen-stimulated primary human T cells. *Blood* **96**, 459–466 (2000). doi:10.1182/blood.V96.2.459 Medline

37. H. Yamaue, S. V. Kashmiri, R. De Filippi, C. Nieroda, J. R. Yannelli, K. Y. Tsang, J. Schlom, Enhanced interleukin-2 production in human tumor-infiltrating lymphocytes engineered by 3′-truncated interleukin-2 gene. *J. Immunother. Emphasis Tumor Immunol.* **16**, 262–274 (1994). doi:10.1097/00002371-199411000-00002 Medline

38. Y. Hart, S. Reich-Zeliger, Y. E. Antebi, I. Zaretsky, A. E. Mayo, U. Alon, N. Friedman, Paradoxical signaling by a secreted molecule leads to homeostasis of cell levels. *Cell* **158**, 1022–1032 (2014). doi:10.1016/j.cell.2014.07.033 Medline

39. Y. Refaeli, L. Van Parijs, C. A. London, J. Tschopp, A. K. Abbas, Biochemical mechanisms of IL-2-regulated Fas-mediated T cell apoptosis. *Immunity* **8**, 615–623 (1998). doi:10.1016/S1074-7613(00)80566-X Medline

40. M. E. Pipkin, J. A. Sacks, F. Cruz-Guilloty, M. G. Lichtenheld, M. J. Bevan, A. Rao, Interleukin-2 and inflammation induce distinct transcriptional programs that promote the differentiation of effector cytolytic T cells. *Immunity* **32**, 79–90 (2010). doi:10.1016/j.immuni.2009.11.012 Medline

41. J. H. Levine, E. F. Simonds, S. C. Bendall, K. L. Davis, A. D. Amir, M. D. Tadmor, O. Litvin, H. G. Fienberg, A. Jager, E. R. Zunder, R. Finck, A. L. Gedman, I. Radtke, J. R. Downing, D. Pe'er, G. P. Nolan, Data-Driven Phenotypic Dissection of AML Reveals Progenitor-like Cells that Correlate with Prognosis. *Cell* **162**, 184–197 (2015). doi:10.1016/j.cell.2015.05.047 Medline

42. M. T. Saung, S. Muth, D. Ding, D. L. Thomas 2nd, A. B. Blair, T. Tsujikawa, L. Coussens, E. M. Jaffee, L. Zheng, Targeting myeloid-inflamed tumor with anti-CSF-1R antibody expands CD137+ effector T-cells in the murine model of pancreatic cancer. *J. Immunother. Cancer* **6**, 118 (2018). doi:10.1186/s40425-018-0435-6 Medline

43. Y. Liu, N. Zhou, L. Zhou, J. Wang, Y. Zhou, T. Zhang, Y. Fang, J. Deng, Y. Gao, X. Liang, J. Lv, Z. Wang, J. Xie, Y. Xue, H. Zhang, J. Ma, K. Tang, Y. Fang, F. Cheng, C. Zhang, B. Dong, Y. Zhao, P. Yuan, Q. Gao, H. Zhang, F. Xiao-Feng Qin, B. Huang, IL-2 regulates tumor-reactive $CD8^+$ T cell exhaustion by activating the aryl hydrocarbon receptor. *Nat. Immunol.* **22**, 358–369 (2021). doi:10.1038/s41590-020-00850-9 Medline

44. S. A. Rosenberg, IL-2: The first effective immunotherapy for human cancer. *J. Immunol.* **192**, 5451–5458 (2014). doi:10.4049/jimmunol.1490019 Medline

45. R. S. Cotran, J. S. Pober, M. A. Gimbrone Jr, T. A. Springer, E. A. Wiebke, A. A. Gaspari, S. A. Rosenberg, M. T. Lotze, Endothelial activation during interleukin 2 immunotherapy. A possible mechanism for the vascular leak syndrome. *J. Immunol.* **140** 1883–1888 (1988). doi:10.4049/jimmunol.140.6.1883 Medline

46. A. Oyler-Yaniv, J. Oyler-Yaniv, B. M. Whitlock, Z. Liu, R. N. Germain, M. Huse, G. Altan-Bonnet, O. Krichevsky, A Tunable Diffusion-Consumption Mechanism of Cytokine Propagation Enables Plasticity in Cell-to-Cell Communication in the Immune System. *Immunity* **46**, 609–620 (2017). doi:10.1016/j.immuni.2017.03.011 Medline

47. K. Thurley, D. Gerecht, E. Friedmann, T. Höfer, Three-Dimensional Gradients of Cytokine Signaling between T Cells. *PLOS Comput. Biol.* **11**, e1004206 (2015). doi:10.1371/journal.pcbi.1004206 Medline

48. H. S. Wong, K. Park, A. Gola, A. P. Baptista, C. H. Miller, D. Deep, M. Lou, L. F. Boyd, A. Y. Rudensky, P. A. Savage, G. Altan-Bonnet, J. S. Tsang, R. N. Germain, A local regulatory T cell feedback circuit maintains immune homeostasis by pruning self-activated T cells. *Cell* **184**, 3981–3997.e22 (2021). doi:10.1016/j.cell.2021.05.028 Medline

49. W. N. D'Souza, L. Lefrançois, IL-2 is not required for the initiation of CD8 T cell cycling but sustains expansion. *J. Immunol.* **171**, 5727–5735 (2003). doi:10.4049/jimmunol.171.11.5727 Medline

50. D. Busse, M. de la Rosa, K. Hobiger, K. Thurley, M. Flossdorf, A. Scheffold, T. Höfer, Competing feedback loops shape IL-2 signaling between helper and regulatory T lymphocytes in cellular microenvironments. *Proc. Natl. Acad. Sci. U.S.A.* **107**, 3058–3063 (2010). doi:10.1073/pnas.0812851107 Medline

51. F. Fuhrmann, T. Lischke, F. Gross, T. Scheel, L. Bauer, K. W. Kalim, A. Radbruch, H. Herzel, A. Hutloff, R. Baumgrass, Adequate immune response ensured by binary IL-2 and graded CD25 expression in a murine transfer model. *eLife* **5**, e20616 (2016). doi:10.7554/eLife.20616 Medline

52. T. Höfer, O. Krichevsky, G. Altan-Bonnet, Competition for IL-2 between Regulatory and Effector T Cells to Chisel Immune Responses. *Front. Immunol.* **3**, 268 (2012). doi:10.3389/fimmu.2012.00268 Medline

53. B. M. Allen, K. J. Hiam, C. E. Burnett, A. Venida, R. DeBarge, I. Tenvooren, D. M. Marquez, N. W. Cho, Y. Carmi, M. H. Spitzer, Systemic dysfunction and plasticity of the immune macroenvironment in cancer models. *Nat. Med.* **26**, 1125–1134 (2020). doi:10.1038/s41591-020-0892-6 Medline
